# Caspase-mediated DDX46 cleavage unchains antiviral immunity

**DOI:** 10.1128/mbio.03519-25

**Published:** 2026-03-19

**Authors:** Yanfeng Liu, Zhongyuan Liu, Xiaoqing Ou, Feiyang Zhao, Jingrui Wang, Yang Qu, Xusheng Qiu, Ying Liao, Lei Tan, Cuiping Song, Chan Ding, Yingjie Sun

**Affiliations:** 1 Shanghai Veterinary Research Institute, Chinese Academy of Agricultural Science 118161 , Shanghai, People's Republic of China; 2 School of Agriculture and Biology, Shanghai Jiao Tong University 12474, Shanghai, China; Albert Einstein College of Medicine, Bronx, New York, USA

**Keywords:** RNA-binding proteins, DDX46, RNA virus, caspase, innate immunity

## Abstract

DEAD/H-box RNA helicases are critical regulators of host antiviral innate immunity. In this study, we utilized an RNA-binding protein knockout sub-library to identify DDX46, a member of the DEAD/H-box RNA helicase family, as an essential proviral host factor for RNA virus replication. While DDX46 has been shown to sequester demethylated innate immune transcripts in the nucleus and dampen interferon (IFN) production, the mechanisms underlying its regulation during viral infection remain unclear. Here, we report that RNA virus infection induces caspase-dependent cleavage of DDX46, triggering its translocation from the nucleus to the cytoplasm. This translocation unchains innate immune transcripts from nuclear retention, licensing their rapid translation and potentiating robust IFN responses. Our findings reveal a novel regulatory mechanism by which post-translational modification and subcellular relocalization of DDX46 fine-tune the host antiviral response, highlighting the functional versatility of RNA helicases in host-virus interactions.

**IMPORTANCE:**

Understanding how host cells regulate innate immune responses to viral infection is essential for developing effective antiviral strategies. Our study uncovers a critical role for caspase-dependent cleavage and nuclear-cytoplasmic translocation of DDX46 in promoting antiviral innate immunity. These findings not only expand our knowledge of the dynamic regulation of DEAD/H-box RNA helicases during infection but also suggest that targeting the post-translational modification and localization of such helicases may offer new avenues for antiviral therapeutic development.

## INTRODUCTION

RNA-binding proteins (RBPs) are a class of proteins that bind specifically to RNA molecules through pocket-like structures on their surfaces (1). RBPs contain multiple RNA-binding domains, such as the RNA recognition motif, zinc finger domains, double-stranded RNA binding domain, K homology domain, and pumilio and FBF homology domain, among others (2, 3). These domains enable RBPs to bind to specific sequences or structural regions in RNA molecules, thereby regulating RNA processing, stability, transport, and translation, as well as other processes essential for cellular functions (4–6). In innate immunity, RBPs play important roles in recognizing pathogen-derived RNA and regulating immune responses. For instance, retinoic acid-inducible gene I (RIG-I) and melanoma differentiation-associated protein 5 (MDA5) detect viral RNA and initiate immune responses by activating downstream signaling pathways, such as NF-κB and IRF3, which promote interferon (IFN) secretion and antiviral responses (7–9). Moreover, RBPs also contribute to the activation of immune cells and the regulation of inflammatory responses. The RNA-binding protein human antigen R (HuR) modulates the stability of specific cytokine mRNAs, thereby influencing the migration and activation of immune cells (10). Studies have shown that HuR enhances the expression of inflammatory cytokines such as TNF-α and IL-6 during inflammation, thereby promoting the occurrence of immune responses (11–13).

DEAD/H-box RNA helicases belong to the superfamily 2 helicase superfamily and are among the largest RNA helicase families found in eukaryotes, bacteria, and even archaea (14). Beyond their well-known role in RNA unwinding, DEAD/H-box helicases are extensively involved in innate immune regulation and viral infections (15). For example, DDX3 counteracts influenza A virus (IAV) by interacting with IAV’s NS1 and NP proteins, which induces the formation of stress granules and inhibits viral replication (16). On the other hand, DDX3 promotes the production of IFN-β through the stimulation of the interferon gene stimulator (STING) pathway (17). Another example is the helicase complex comprising DDX1, DDX21, and DHX36, which interacts with dsRNA and the adaptor protein TIR-domain-containing adapter-inducing interferon-β (TRIF), activating downstream IRF3/7 pathways and promoting IFN production independent of Toll-like receptor 3 (18, 19).

DDX46 belongs to the DEAD-box RNA helicase family, with its yeast homolog being Prp5p. Both are crucial for precursor mRNA (pre-mRNA) splicing (20). In eukaryotes, pre-mRNA must undergo intron removal and exon ligation to form mature mRNA (21). During early spliceosome assembly, DDX42 binds to splicing factor 3B and recruits the 12S U2 snRNP, forming the DDX42-U2 complex. At this point, DDX46 competitively binds to the complex, leading to the dissociation of DDX42. This process marks the maturation of the 17S U2 snRNP (20). In addition to its role in spliceosome assembly, DDX46 plays an important role in the cell cycle and the development of cancer. Studies have shown that DDX46 is crucial in cell division; *DDX46* knockdown induces apoptosis and causes cell cycle arrest at the G1 phase, thereby inhibiting cell proliferation and colony formation (22). Furthermore, other studies have demonstrated that DDX46 contributes to the proliferation of cancer cells. DDX46 activates the mitogen-activated protein kinase p38 (MAPK-p38) pathway, thereby promoting the proliferation of glioblastoma multiforme cells (23). However, research on the role of DDX46 in innate immunity and viral replication remains limited. A recent study has revealed that DDX46 interacts with the transcripts of innate immune molecules, including mitochondrial antiviral signaling protein (MAVS), tumor necrosis factor receptor-associated factor (TRAF) 3, and TRAF6, through its conserved element CCGGUU. Through its helicase domain, DDX46 recruits the m6A demethylase ALKBH5, which demethylates these transcripts, retaining them in the nucleus and inhibiting their translation and IFN production (24).

In this study, we conducted a CRISPR-Cas9 knockout (KO) screen targeting a subset of RBP genes and identified DDX46 as a key member of the DEAD/H-box helicase family that plays a key role in regulating viral replication. Our results demonstrated that *DDX46* KO significantly inhibits the replication of Newcastle disease virus (NDV) and vesicular stomatitis virus (VSV). Furthermore, we found that RNA viruses and RNA ligands induce caspase-mediated cleavage of DDX46 at the aspartate 226 (D226) site, resulting in its translocation from the nucleus to the cytoplasm. In the cytoplasm, the cleaved DDX46 loses its ability to suppress the nuclear export of innate immune transcripts, such as MAVS and TRAF3, thereby enhancing the antiviral immune response. Overall, our findings uncover the role of post-translational modification of DDX46 in regulating innate immunity during RNA virus infection, providing new insights into the mechanisms of host antiviral immune regulation.

## MATERIALS AND METHODS

### Cell cultures and virus

The cell lines HeLa, H1299, HEK293T, A549, Vero, and BHK21 were obtained from the American Type Culture Collection. HeLa, HEK293T, Vero, and BHK21 cells were maintained in Dulbecco’s modified Eagle’s medium, while H1299 and A549 cells were cultured in RPMI‐1640 medium, both supplemented with 10% fetal bovine serum (Thermo Fisher Scientific), at 37°C in a 5% CO_2_ atmosphere.

Herpes simplex virus (HSV)-1 was provided by Yasushi Kawaguchi (University of Tokyo, Japan), and VSV and GFP-VSV were provided by Jianchao Wei (Shanghai Veterinary Research Institute, China). NDV Herts/33 strain was obtained from the China Institute of Veterinary Drug Control (Beijing, China). Recombinant fluorescent-tagged virus NDV La Sota strain (RFP-NDV) was provided by Zengqi Yang (Northwest A&F University, China). The titers of HSV-1 and VSV were measured as the median tissue culture infectious dose (TCID_50_) using Vero cells, while the titer of NDV was determined as the TCID_50_ using BHK21 cells.

### Reagents and antibodies

The caspase inhibitor Z-VAD-FMK (C1202, Beyotime) was utilized at a concentration of 50 μM. The neddylation inhibitor MLN4924 (S7109, Selleckchem) was utilized at 1 μM, and the proteasome inhibitor MG-132 (S1748, Beyotime) was utilized at 20 μM. Autophagy inhibitors wortmannin (W1628, Sigma-Aldrich) and chloroquine (CQ) (C6628, Sigma-Aldrich) were utilized at concentrations of 300 nM and 25 μM, respectively. poly(I:C) (tlrl-pic), 3p-hpRNA (tlrl-hprna), HSV-60 (tlrl-hsv60n), and poly(G:C) (tlrl-pgcn) were all obtained from Invivogen.

The mouse monoclonal anti-Flag antibody (F1804) was purchased from Sigma-Aldrich. The rabbit polyclonal anti-DDX46 antibody (ab72083) and rabbit polyclonal anti-VSV-G antibody (ab1874) were purchased from Abcam. The rabbit polyclonal anti-HSV-1 antibody (NB600-516) was obtained from Novus Biologicals. The rabbit monoclonal anti-β-actin antibody (AC006) was purchased from ABclonal. The rabbit monoclonal anti-TBK1/NAK (38066), rabbit monoclonal anti-phospho-TBK1/NAK (Ser172) (D52C2), and rabbit monoclonal anti-Lamin B1 (13435) antibodies were purchased from Cell Signaling Technology. The mouse monoclonal anti-β-tubulin antibody (AC021) was purchased from ABclonal.

The cytoplasmic and nuclear RNA purification kit (NBS21000) was purchased from Shanghai Qinxin Biotechnology. The human MAVS and TRAF3 RNA probes were custom-designed by Bioengineering (Shanghai).

### Plasmids

Flag-DDX46-X1 and -X2 were constructed by inserting the open reading frames of human DDX46 isoform 1 (NM_001300860.2) and isoform 2 (NM_014829.4) into plasmid p3×FLAG-CMV-14 (Sigma-Aldrich), respectively. Flag-tagged deletion constructs and point mutants of DDX46 were generated by site-directed mutagenesis, as described previously (19). The WT-DDX46 and D226A-DDX46 containing an HA tag at the C-terminus were cloned into the pHAGE-bsd plasmid, which was constructed based on pHAGE-puro (118692, Addgene). The primers are detailed in Table S1.

### SDS-PAGE and western blot analysis

Total protein was extracted by lysing cells on ice using RIPA lysis buffer (P0013D, Beyotime) supplemented with protease and phosphatase inhibitors (PR20015, Proteintech). The lysates were denatured at 100°C for 10 min and subsequently subjected to SDS-PAGE for protein separation. Proteins were then transferred onto nitrocellulose membranes and blocked with 5% skim milk for 1 h. The membranes were incubated with primary antibodies at 4°C overnight, followed by incubation with horseradish peroxidase-conjugated secondary antibodies at room temperature for 1 h. Protein bands were visualized using an ECL detection kit (PK10003, Proteintech).

### RNA extraction and qRT-PCR

The total cellular RNA was extracted using the MolPure Cell RNA Kit (19231ES50, Yeasen). In general, 400 µL of lysis buffer LB was added per 1 × 10⁶ cells, followed by lysis for 1 min at room temperature. The lysate was then collected, and the subsequent steps were carried out according to the manufacturer’s instructions. Finally, RNA was dissolved in and collected using 500 µL of RNase-free water. For nuclear and cytoplasmic RNA separation, the Nuclear-Cytoplasmic Fractionation Kit (21000, Norgen Biotek) was used to extract cytoplasmic and nuclear RNA. cDNA synthesis was performed using the Hifair II 1st Strand cDNA Synthesis Kit (11121ES60, Yeasen). The reaction conditions were as follows: 25°C for 5 min, 42°C for 30 min, and 85°C for 5 min. The relative mRNA levels were calculated using the ΔΔCT method and normalized to GAPDH mRNA. The primer sequences are shown in Table S1.

### RNA interference

The small interfering RNA (siRNA) oligonucleotides were provided by Shanghai GenePharma (A01001). Cells were transfected with the specified siRNA or control siRNA as described previously (25). At 48 h post-transfection (hpt), the knockdown efficiency of the target gene was validated by quantitative real-time polymerase chain reaction (RT-qPCR) or western blot. The siRNA sequences are listed in Table S1.

### Generation of CRISPR library and lentivirus

In this study, a human RBP pooled CRISPR KO library (1,078 RBP genes, 141438, Addgene) was utilized to identify RBPs that promote NDV replication in A549 cells. Briefly, the sgRNA plasmid library, psPAX2 (12260, Addgene), and pMD2.G (12259, Addgene) were co-transfected into HEK293T cells (T225 flasks, 70%–80% confluence) at a ratio of 15.3 µg:23.4 µg:30.6 µg. Approximately 48 hpt, the supernatant containing lentivirus was harvested, clarified by centrifugation at 3,500 rpm for 20 min, and stored at −80°C. The lentiviral supernatant was subsequently used to infect A549 cells at a low multiplicity of infection (MOI = 0.3) to ensure that each cell incorporated no more than one sgRNA. After 48 h post-infection (hpi), puromycin (1 mg/mL) was added to the culture to eliminate uninfected cells, resulting in the generation of a pooled CRISPR RBP library in A549 cells.

### CRISPR screening of RBPs for NDV replication

We performed a pooled CRISPR KO screen in A549 cells using a lethal, high-MOI RFP-NDV challenge to select surviving clones and identify RBPs critical for NDV replication. For the successive CRISPR screening to identify RBPs critical for NDV replication, CRISPR library-transduced A549 cells (1 × 10^8^) were subjected to high-MOI (MOI = 3) infection with RFP-NDV for 72 h. Following the viral challenge, surviving cells were expanded until the population recovered to 1 × 10⁸ cells. To enrich for resistant clones, this cell pool underwent two rounds of stringent RFP-NDV infection under identical conditions. Genomic DNA was extracted from the uninfected cells or the cells that survived NDV infection, and sgRNA sequences were amplified and subjected to next-generation sequencing using an Illumina HiSeq instrument. Sequencing data were enriched and analyzed using the MAGeCK package for the sgRNA database, where genes with a *P*-value less than 0.05 were considered significant. Members of the DDX/DHX family were selected for further analysis (the enrichment of sgRNA for DDX/DHX family members and other RBPs is shown in Table S2). GraphPad Prism software was used to load and visualize the output graphs.

### Generation of *DDX46* KO cells

The CRISPR-Cas9 system was used to knock out the target gene. Briefly, the sgRNAs targeting *DDX46* were designed using an online tool, Synthego (https://design.synthego.com; sgRNA: TCGAAAACGATCGGCATCCC) and cloned into the LentiCRISPR-v2 plasmid (containing Cas9 and a puromycin selection marker). The constructed plasmid, along with psPAX2 and pMD2.G packaging plasmids, was transfected into the target cell line using Lipofectamine 2000 (11668019, Thermo Fisher Scientific). After 48 h, cells were selected in puromycin-containing medium (1 mg/mL) to enrich successfully transfected cells. Following 48 h of selection, cells were subjected to single-cell cloning via dilution plating. After clonal expansion, western blotting and gene sequencing were performed to validate the knockout efficiency of the target gene.

### Generation of cells stably expressing wild-type (WT) and mutant DDX46

HEK293T cells were co-transfected with pHAGE-WT- and -D226A-DDX46 plasmids, along with two packaging plasmids, pMD2.G and psPAX2. At 60 hpt, the cell supernatants were collected, centrifuged at 5,000 rpm for 10 min, and filtered. The lentivirus-containing supernatants, supplemented with 5 mg/mL Polybrene (H9268-50G, Sigma-Aldrich), were added to *DDX46*^+/-^ HeLa cells. After 48 hpi, the supernatants were replaced with culture medium containing 10% FBS, and the cells were cultured for an additional 72 h. The expression efficiency was confirmed by immunoblotting.

### Indirect immunofluorescence analysis

Cells were washed with Tris-Buffered Saline with Tween 20 (TBST) and fixed with 4% paraformaldehyde for 10 min, followed by permeabilization with 0.5% Triton X-100 for 10 min. The cells were then incubated in blocking buffer (3% BSA in PBS) for 1 h. After blocking, the cells were incubated with a specific primary antibody at 37°C for 3 h. Following three washes with TBST, the cells were incubated with a fluorescently conjugated secondary antibody for 1 h. Subsequently, the cells were washed again and stained with 0.5 mg/mL 4′,6-diamidino-2-phenylindole (DAPI). Subcellular localization of the target protein was examined using a Zeiss LSM880 confocal microscope.

### RNA sequencing and analysis

RNA library construction and high-throughput sequencing were performed by Wuhan Mettewell Technology. In brief, total RNA was extracted from cells using TRIzol reagent, and library construction was completed using the Illumina Stranded mRNA Prep Ligation platform. High-throughput sequencing was conducted on the NovaSeq X Plus platform. Differential expression analysis was performed using DESeq2 software, with the default criteria for identifying significantly differentially expressed genes set at FDR < 0.05 and |log_2_FC| ≥ 1. A gene is considered differentially expressed when it simultaneously meets both conditions.

### Fluorescent *in situ* hybridization (FISH)

FISH was used to detect the expression and localization of MAVS and TRAF3 mRNAs in cells. Probes were synthesized by Sangon Biotech with the following sequences: MAVS probe: 5′-Cy3-CCAAAGGUGCCCUCGGACUUAUACUCAUUCUCCUCU-3′; TRAF3 probe: 5′-Cy3-UAGGCGGAUGUCGUGCACACUCAGCAUCUGGUCAU-3′. Cells were cultured on glass slides and fixed with 4% paraformaldehyde for 15 min, followed by PBS washing. Cells were then permeabilized with 0.1% Triton X-100 at room temperature for 10 min. Pre-hybridization was carried out in pre-hybridization buffer at 37°C for 30 min. Probes (100 nM) were added to the hybridization buffer, and hybridization was performed overnight at 37°C in a humidified chamber. After hybridization, samples were washed at 42°C with 4× saline sodium citrate buffer (SSC), 2× SSC, and 1× SSC to remove unbound probes. Nuclei were counterstained with DAPI, and slides were mounted using antifade mounting medium. ImageJ software (NIH, USA) was utilized to quantify the distribution intensity of MAVS or TRAF3 mRNA signals within the nucleus and cytoplasm.

### Isolation of nuclear and cytoplasmic proteins

For the separation and sample preparation of nuclear and cytoplasmic proteins, the Nuclear and Cytoplasmic Protein Extraction Kit (P0027) from Beyotime was utilized following the manufacturer’s instructions. Briefly, cells were first resuspended in cytoplasmic extraction buffer A containing phenylmethylsulfonyl fluoride, vigorously vortexed, and incubated on ice, followed by the addition of cytoplasmic extraction buffer B and centrifugation to collect the cytoplasmic fraction. The pellet was then resuspended in nuclear extraction buffer, vortexed intermittently during incubation, and centrifuged to obtain the nuclear protein extract.

### Statistical analysis

Statistical analyses were performed using GraphPad Prism software. Data were expressed as means ± standard deviations. Significance was determined with the two-tailed independent Student’s *t*-test (*P* < 0.05) between two groups. A one-way ANOVA was followed by Tukey’s test to compare multiple groups (>2).

## RESULTS

### DDX46 facilitates NDV and VSV replication while negatively regulating innate immunity

**Fig 1:**
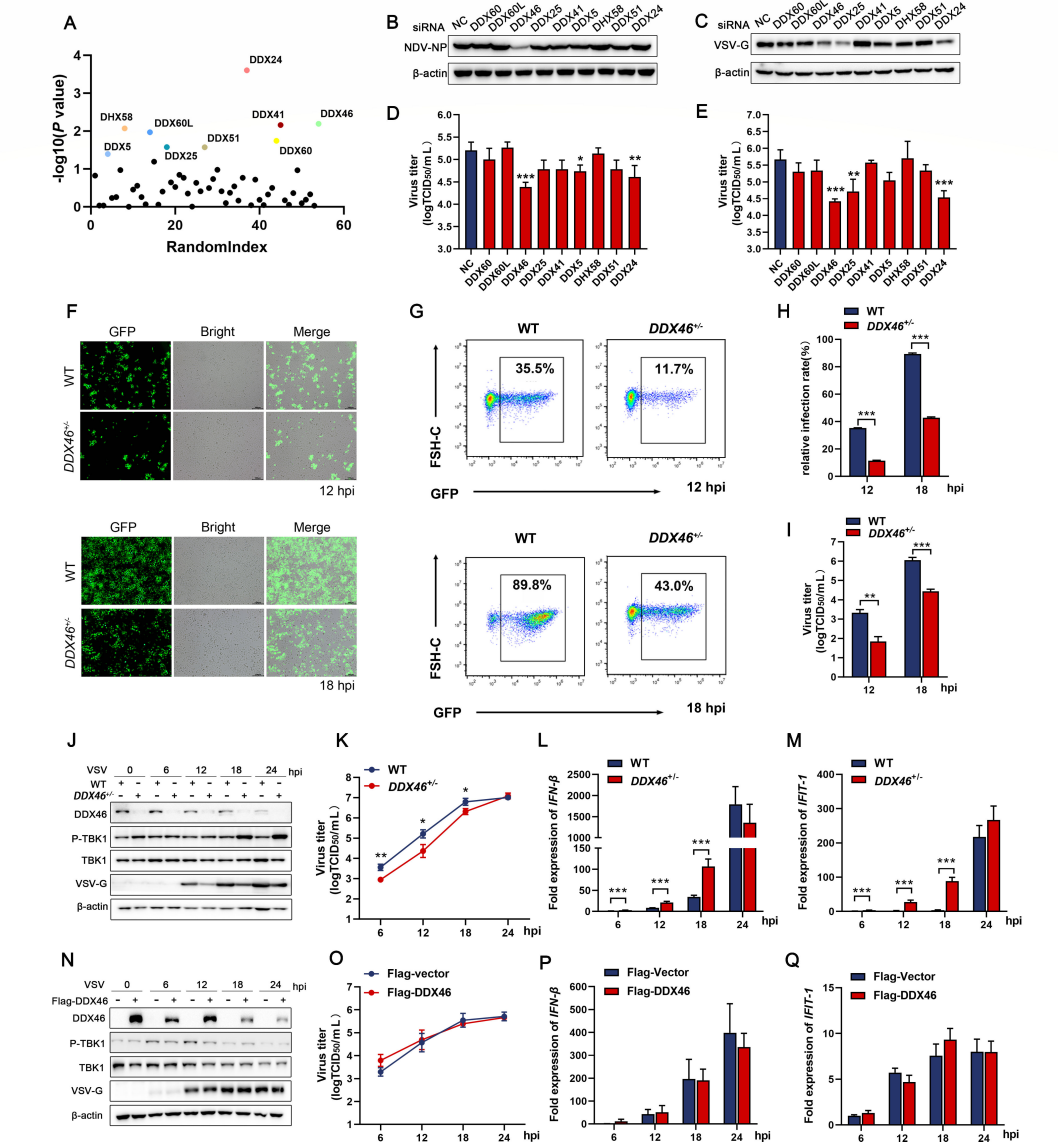
DDX46 negatively regulates the IFN-β signaling pathway. (**A**) Pooled CRISPR screen identifies DDX/DHX helicases that modulate NDV replication. A549 cells transduced with an RBP-targeted CRISPR knockout library (~1 × 10^8^) underwent two rounds of lethal, high-MOI RFP-NDV challenge (MOI = 3, 72 h), with expansion between rounds. sgRNAs from uninfected controls and survivors were PCR-amplified and quantified by next-generation sequencing. The plot displays all DDX/DHX family members, with nine prioritized candidates highlighted in color based on the highest −log_10_(*P*-value <0.05), and the remaining genes shown in black. *X*-axis: gene count and RandomIndex. (**B, C**) A549 cells were transfected with siNC or siRNAs targeting the nine DDX/DHX candidates for 48 h, then infected with NDV (**B**) or VSV (**C**) at an MOI of 0.1 for 12 h, followed by western blot (WB) analysis of NDV NP and VSV-G. β-Actin served as the loading control. (**D, E**) Virus infection experiments were performed as in panels **B and C**. Extracellular viral titers were determined by TCID_50_ for NDV (**D**) and VSV (**E**). (**F–I**) WT and *DDX46*^+/-^ HeLa cells were mock-infected or infected with GFP-VSV (MOI = 0.1) for 12 and 18 h, followed by imaging using GFP and brightfield channels (**F**). Flow cytometry analysis with GFP on the *x*-axis and FSC-H on the *y*-axis (**G**), and quantification of relative infection rate (%) based on the flow cytometry data (**H**). Extracellular GFP-VSV titers were determined as TCID_50_ on Vero cells (**I**). (**J–M**) WT and *DDX46*^+/-^ HeLa cells were mock-infected or infected with VSV (MOI=1) for 6, 12, 18, and 24 h. Protein levels of DDX46, p-TBK1, TBK1, and VSV-G were analyzed by WB. β-Actin served as the loading control (**J**). Extracellular VSV titers were determined by TCID_50_ (**K**). The intracellular mRNA levels of IFN-β (**L**) and IFIT-1 (**M**) were analyzed using qRT-PCR. (**N–Q**) HeLa cells were transfected with the empty vector p3×Flag or Flag-DDX46 for 24 h, then mock infected or infected with VSV (MOI = 1) for 6, 12, 18, and 24 h. Protein levels of DDX46, p-TBK1, TBK1, and VSV-G were analyzed by WB. β-Actin served as the loading control (**N**). Extracellular VSV titers were determined by TCID_50_ (**O**). The intracellular mRNA levels of IFN-β (**P**) and IFIT-1 (**Q**) were analyzed using qRT-PCR. Data are presented as means from three independent experiments. *P < 0.05, **P < 0.01, ***P < 0.001.

RBPs constitute a key interface of virus-host interactions, particularly for RNA viruses. To define host determinants of NDV replication, we constructed an RBP pooled CRISPR knockout screen and performed two rounds of stringent viral screening. Surviving cells were expanded and subjected to high-throughput sequencing. The screen yielded hits from multiple RBP families (ribosomal/translation, RNA splicing/processing, nuclear transport, nucleolar/ribosome biogenesis, etc.; Table S2). Building on our prior work and the recognized roles of DEAD/H-box helicases in RNA virus infection, we prioritized DDX/DHX candidates for follow-up. Based on the screening results, we highlighted nine DDX/DHX genes selected by their highest −log_10_(*P*-value) for follow-up validation (Fig. 1A). These prominent DDX/DHXs were selected for individual knockdown experiments, followed by infection with NDV and VSV. The results showed that *DDX46* knockdown significantly inhibited viral protein expression and reduced the viral titers in the cell supernatant for both viruses (Fig. 1B through E). Next, we generated *DDX46* knockout cells, identifying two heterozygous clones (*DDX46*^+/-^ #1 and *DDX46*^+/-^ #2, Fig. S1A). Western blotting confirmed reduced DDX46 expression (Fig. S1B). Microscopy (Fig. 1F) and flow cytometry (Fig. 1G and H) showed a significant reduction in VSV-GFP replication in DDX46 KO (*DDX46*^+/-^ #1) cells compared with WT cells, consistent with the extracellular virus titers (Fig. 1I). Furthermore, time-course experiments revealed that, following VSV and NDV infection, *DDX46* KO (*DDX46*^+/-^ #1) significantly decreased viral protein synthesis (Fig. 1J; Fig. S1C) and the viral titers in the supernatant (Fig. 1K; Fig. S1D), consistent with the effects observed in DDX46 knockdown cells. Additionally, *DDX46* KO significantly enhanced the phosphorylation of TBK1, the key molecule in antiviral innate immunity signaling (Fig. 1J; Fig. S1C). Accordingly, mRNA levels of IFN-β and its downstream interferon-stimulated gene (ISG), interferon-induced protein with tetratricopeptide repeats 1 (IFIT-1), were significantly upregulated in *DDX46*^+/-^ cells compared to WT cells following VSV and NDV infection (Fig. 1L and M; Fig. S1E and F). Similar results were observed in *DDX46*^+/-^ #2 cells (Fig. S1G through J). Furthermore, in murine macrophage RAW264.7 cells, *Ddx46* knockdown suppressed VSV replication while enhancing IFN-β expression (Fig. S1K through M). Interestingly, although *DDX46* knockdown strongly influenced viral replication and antiviral signaling, no significant changes were observed in DDX46-overexpression cells upon VSV infection (Fig. 1N through Q). Taken together, these findings indicate that DDX46 facilitates NDV and VSV replication while serving as a negative regulator of innate immunity. However, the inconsistency between the knockdown and overexpression models suggests that, beyond DDX46 expression, other regulatory mechanisms involving DDX46 may contribute to the regulation of antiviral innate immunity.

### DDX46 protein levels are downregulated by RNA viruses and RNA ligands

**Fig 2:**
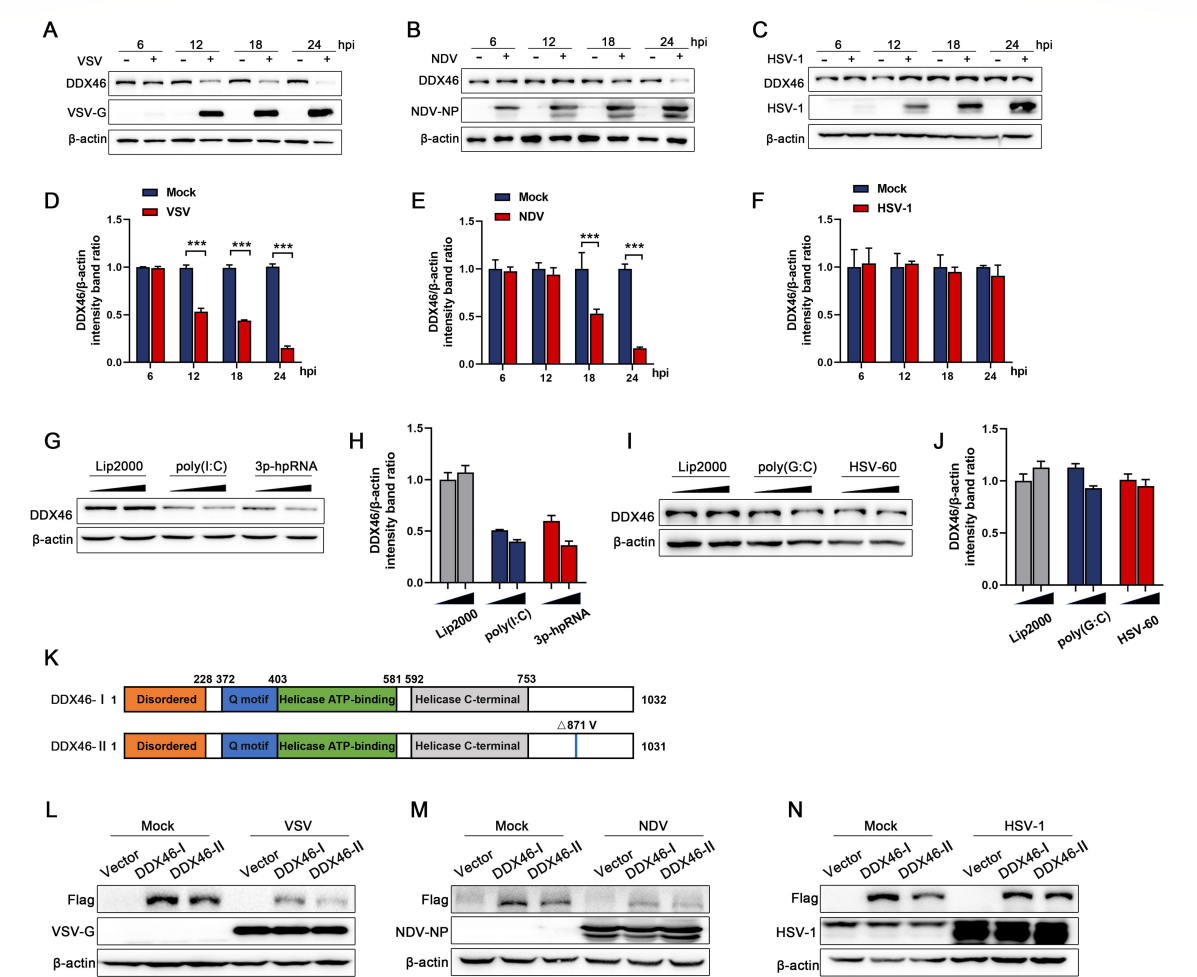
Virus infection or treatment with RNA ligands induces reduced DDX46 protein levels. (**A–C**) HeLa cells were mock-infected or infected with VSV (**A**), NDV (**B**), or HSV-1 (**C**) at an MOI of 1 for 6, 12, 18, and 24 h. Protein levels of VSV-G, NDV-NP, or HSV-1-gD were analyzed by WB. β-Actin served as the loading control. (**D–F**) Representative results, with graphs representing the band intensity ratios of DDX46/β-actin normalized to the control conditions for the VSV (**D**), NDV (**E**), and HSV-1 (**F**) infection groups. (**G–J**) HeLa cells were transfected with RNA ligands [poly(I:C) or 3p-hpRNA, panels** G and H**] or DNA ligands [poly(G:C) or HSV-60, panels **I and J**] for 18 h. Protein levels of DDX46 were analyzed by WB. β-Actin served as the loading control. Representative results, with graphs representing the band intensity ratios of DDX46/β-actin normalized to the control conditions for the RNA ligands (**H**) or DNA ligand treatment groups (**J**). (**K**) Schematic diagram of DDX46 isoform I (full-length; DDX46-I) and isoform II (lacking valine at amino acid 872; DDX46-II). (**L–N**) HeLa cells were transfected with the empty vector p3×Flag, Flag-DDX46-I, or Flag-DDX46-II for 24 h, then mock infected or infected with VSV (**L**), NDV (**M**), or HSV-1 (**N**) at an MOI of 1 for 6, 12, 18, and 24 h. Protein levels of exogenous Flag-DDX46 and viral proteins (VSV-G, NDV-NP, or HSV-1-gD) were analyzed by WB. β-Actin served as the loading control. Data are presented as means from three independent experiments. ***P < 0.001.

To further examine whether viral infection regulates DDX46 protein expression, HeLa cells were infected with two RNA viruses (NDV and VSV) and one DNA virus (HSV-1). The results revealed that DDX46 protein levels progressively decreased over time following infection with VSV and NDV, whereas no significant changes were observed with HSV-1 infection (Fig. 2A through F). To rule out cell-specific effects, A549 and H1299 cells were chosen for verification. Similar to the results in HeLa cells, NDV and VSV significantly reduced DDX46 protein levels, while HSV-1 had no effect (Fig. S2). DDX46, as an RNA helicase, is capable of binding to and recognizing various types of RNA. Based on this property, we hypothesized that the observed reduction in DDX46 protein levels following RNA virus infection may stem from its interactions with viral nucleic acids. This interaction could potentially explain why DNA viruses fail to induce a similar response. To test this hypothesis, HeLa cells were stimulated with two RNA ligands [poly(I:C) and 3p-hpRNA] and two DNA ligands [poly(G:C) and HSV-60]. Consistent with our hypothesis, only RNA ligands, and not DNA ligands, triggered a downregulation of DDX46 protein levels (Fig. 2G through J). DDX46 exists in two isoforms: isoform I (full-length) and isoform II (lacking valine at amino acid 872) (Fig. 2K). To test their response to RNA versus DNA viruses, we assessed the protein levels of both isoforms after viral infection. Both were significantly downregulated by RNA viruses (NDV and VSV) but not by the DNA virus (HSV-1) (Fig. 2L through N). Taken together, these results indicate that RNA viruses and RNA ligands can specifically induce the downregulation of DDX46 protein expression.

### DDX46 was cleaved at D226 in a caspase-dependent manner

**Fig 3:**
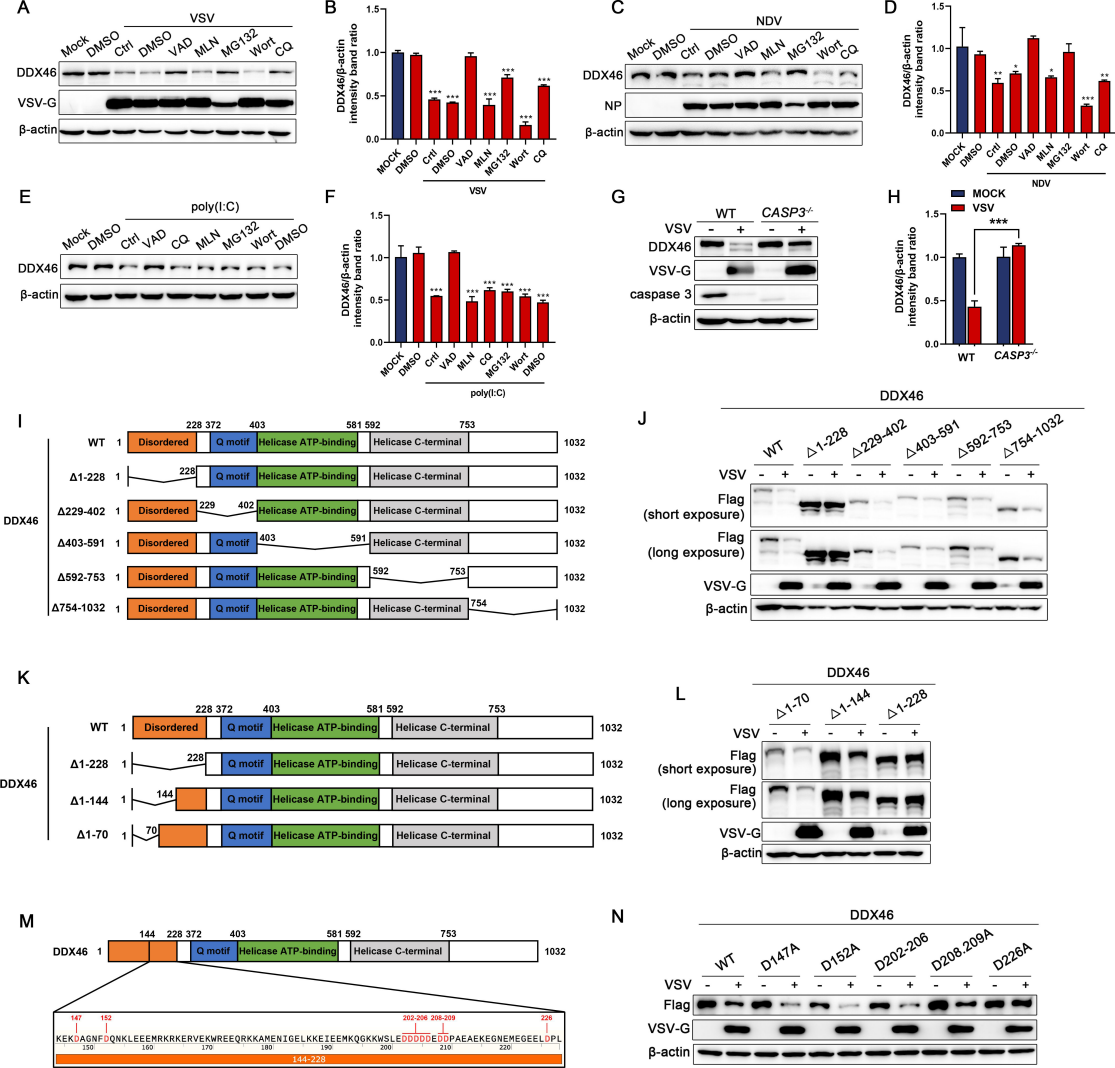
DDX46 undergoes caspase-dependent cleavage at Asp226. (**A–D**) HeLa cells were mock-infected or infected with VSV (**A, B**) or NDV (**C, D**) at an MOI of 1 and maintained in the presence of dimethyl sulfoxide (DMSO) control, z-VAD-FMK (VAD), MLN4924 (MLN), MG-132, wortmannin (Wort), or CQ for 18 h. Protein levels of DDX46 and viral proteins (VSV-G or NDV-NP) were analyzed by WB. β-Actin served as the loading control. Representative results, with graphs representing the band intensity ratios of DDX46/β-actin normalized to the control conditions for VSV (**B**) or NDV (**D**) infection groups. (**E, F**) HeLa cells were transfected with poly(I:C) and maintained in the presence of the DMSO, VAD, CQ, MLN, MG-132, Wort, or CQ for 18 h. Protein levels of DDX46 were analyzed by WB. β-Actin served as the loading control (E). Representative results, with graphs representing the band intensity ratios of DDX46/β-actin normalized to the control conditions (**F**). (**G, H**) WT and *CASP3*^-/-^ HeLa cells were mock-infected or infected with VSV (MOI = 1) for 18 h. Protein levels of DDX46, VSV-G, and caspase 3 were analyzed by WB. β-Actin served as the loading control (**G**). Representative results, with graphs representing the band intensity ratios of DDX46/β-actin normalized to the control conditions (**H**). (**I**) Schematic diagram of WT-DDX46 and truncation/deletion mutants (Δ1–228, Δ229–402, Δ403–591, Δ592–753, and Δ754–1,032). (**J**) HeLa cells were transfected with Flag-tagged WT-DDX46 or truncation/deletion mutants (DDX46 Δ1–228, Δ229–402, Δ403–591, Δ592–753, and Δ754–1,032), then infected with VSV (MOI = 1) for 18 h. Protein levels of exogenous Flag-DDX46 and VSV-G were analyzed by WB. β-Actin served as the loading control. (**K**) Schematic diagram of WT-DDX46 and N-terminal disordered-region truncation mutants (DDX46 Δ1–70, Δ1–144, and Δ1–228). (**L**) HeLa cells were transfected with Flag-tagged WT-DDX46 or truncation mutants (Δ1–70, Δ1–144, and Δ1–228), then infected with VSV (MOI = 1) for 18 h. Protein levels of exogenous Flag-DDX46 and VSV-G were analyzed by WB. β-Actin served as the loading control. (**M**) Schematic of aspartic acid (D) residues mapped within the DDX46 region identified to mediate cleavage (aa 144–228). (**N**) HeLa cells were transfected with Flag-tagged WT-DDX46 or point mutants (D147A, D152A, D202–206A, D208.209A, D226A), then infected with VSV (MOI = 1) for 18 h. Protein levels of exogenous Flag-DDX46 and VSV-G were analyzed by WB. β-Actin served as the loading control. Data are presented as means from three independent experiments. *P < 0.05, **P < 0.01, ***P < 0.001.

To investigate the mechanisms underlying the downregulation of DDX46 protein levels, HeLa cells were infected with NDV and VSV, followed by treatment with various inhibitors, including the caspase inhibitor (Z-VAD-FMK), proteasome inhibitor (MG132), neddylation inhibitor (MLN4924), and autophagy inhibitors (wortmannin and chloroquine). Western blot and statistical analyses showed a significant restoration of DDX46 protein levels following treatment with Z-VAD-FMK and MG132 (Fig. 3A through D). Notably, the strong inhibitory effect of MG132 on viral replication suggests that its regulation of DDX46 may be a secondary effect. To rule out interference with viral replication, HeLa cells were stimulated with poly(I:C) and treated with inhibitors. Only Z-VAD-FMK significantly restored DDX46 protein levels (Fig. 3E and F). Additionally, in *CASP3* KO cells infected with VSV, DDX46 protein levels were significantly higher compared to infected WT cells (Fig. 3G and H). These results strongly suggest that the reduction in DDX46 protein levels caused by RNA viruses and RNA ligands is primarily due to caspase-dependent cleavage. The structure of DDX46 primarily comprises four regions: the N-terminal disordered sequence (1–228 aa), the Q motif (372–403 aa), the helicase ATP-binding domain (592–753 aa), and the helicase C-terminal domain (754–1,032 aa). To further determine the cleavage site of DDX46, six domain deletion mutants of DDX46 were constructed: DDX46 ∆1-228, ∆229-402, ∆403-591, ∆592-753, and ∆754-1032 (Fig. 3I). The results showed that deletion of the N-terminal disordered sequence (1–228 aa) significantly inhibited the cleavage of DDX46 upon VSV infection (Fig. 3J). To further pinpoint the cleavage target of DDX46, two truncation mutants targeting the N-terminal disordered region were constructed: DDX46 ∆1-70 and ∆1-144 (Fig. 3K). Compared to these mutants, cleavage of DDX46 ∆1-228 was significantly inhibited following VSV infection, suggesting that the cleavage site lies within 145–228 aa (Fig. 3L). Given that the cleavage site for the Caspase protein family is aspartic acid (Asp [D]), all the Asp residues within 145–228 aa of DDX46 were mutated to alanine (Ala [A]) (Fig. 3M). The results showed that viral infection experiments revealed that the D226A mutation significantly inhibited DDX46 cleavage (Fig. 3N). Collectively, these results indicated that virus infection leads to the cleavage of DDX46 at D226 in a caspase-dependent manner.

### Viral infection induces DDX46 cleavage and translocation from the nucleus to the cytoplasm

**Fig 4:**
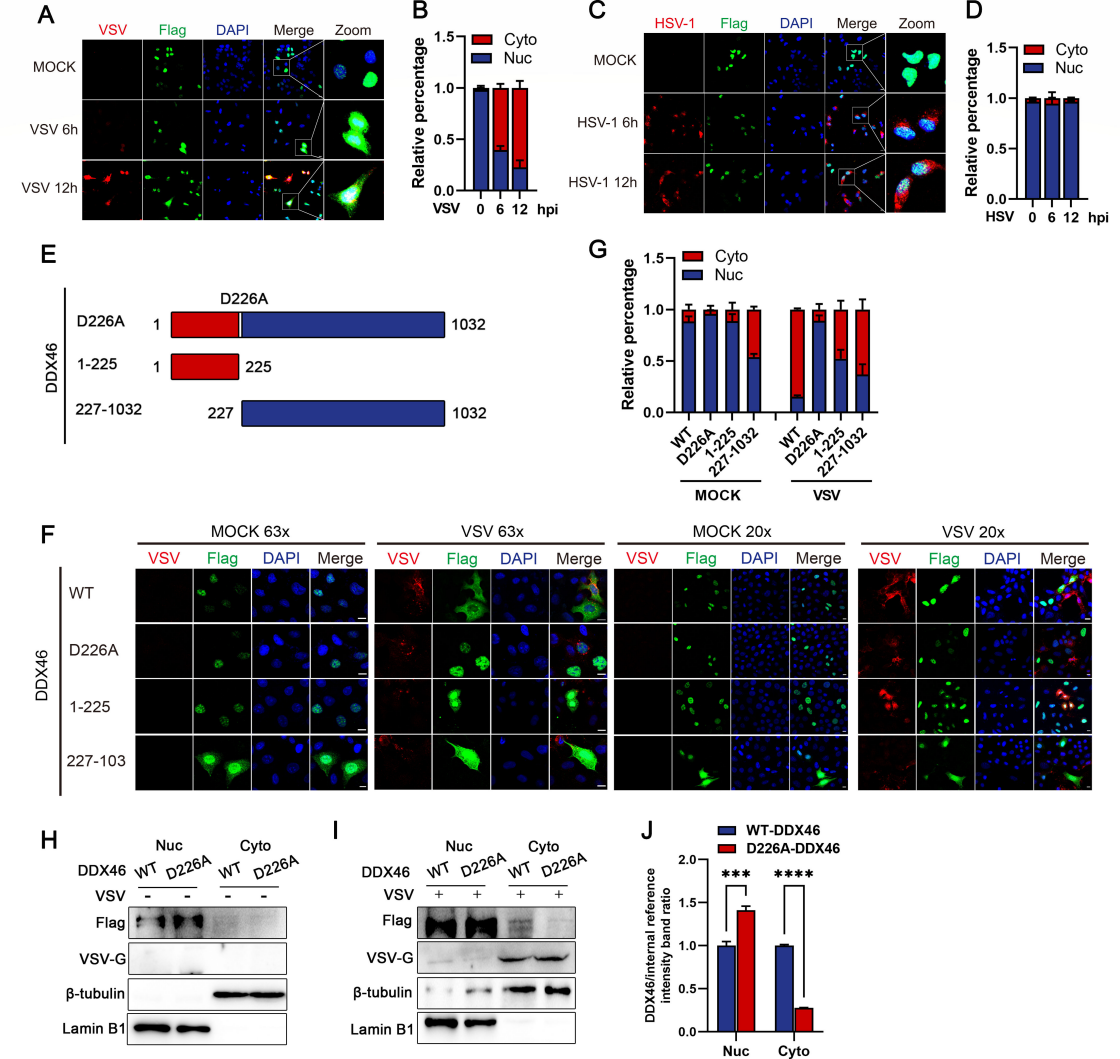
Viral infection induces DDX46 cleavage and translocation from the nucleus to the cytoplasm. (**A–D**) HeLa cells were transfected with the Flag-DDX46 for 24 h, then mock-infected or infected with VSV (**A, B**) or HSV-1 (**C, D**) at an MOI of 1 for 6 and 12 h. Cells were fixed and subjected to IF analysis using anti-Flag and anti-viral-protein (VSV-G or HSV-1-gD) antibodies. Nuclei were counterstained with DAPI. Quantification of the relative percentages of cells with nuclear (Nuc) and cytoplasmic (Cyto) Flag signal after VSV (**B**) or HSV-1 (**D**) infection. Six randomly selected fields were analyzed using ImageJ. (**E**) Schematic of DDX46 mutants: point mutant D226A, N-terminal truncation (1–225), and C-terminal truncation (227–1,032). (**F, G**) HeLa cells were transfected with Flag-tagged WT-DDX46 or mutants (D226A, 1–225, and 227–1,032) for 24 h, then mock-infected or infected with VSV (MOI = 1) for 12 h. Cells were fixed and subjected to IF analysis using anti-Flag and anti-VSV-G antibodies. Nuclei were counterstained with DAPI. The two panels on the left show wider fields at 63×, and the two panels on the right show smaller fields at 20× (**F**). Quantification of the relative percentages of cells with nuclear or cytoplasmic Flag signal after VSV infection. Six randomly selected fields were analyzed using ImageJ (**G**). (**H and I**) HeLa cells were transfected with Flag-tagged WT-DDX46 or D226A-DDX46 for 24 h, then mock-infected (**H**) or infected with VSV at an MOI of 1 (**I**) for 12 h. Cells were harvested, and nuclear and cytoplasmic fractions were prepared using a nucleocytoplasmic isolation kit. Protein levels of exogenous Flag-DDX46 and VSV-G were analyzed by WB. β-Tubulin and Lamin B1 served as the loading controls for cytoplasmic and nuclear fractions, respectively. (**J**) Representative results, with graphs showing the band intensity ratios of DDX46/β-actin normalized to the control conditions for the VSV infection group (**I**). Data are presented as means from three independent experiments. ***P < 0.001.

It is noteworthy that many helicases, such as heterogeneous nuclear ribonucleoprotein M, glioma tumor suppressor candidate region gene 2 protein, and DDX21, translocate from the nucleus to the cytoplasm following viral infection, thereby influencing downstream innate immune responses (19, 26, 27). To examine DDX46 localization during viral infection, HeLa cells were infected with VSV and HSV-1, and the localization of DDX46 was analyzed via immunofluorescence. Under physiological conditions, DDX46 is confined to the nucleus. During VSV infection, it translocates to the cytoplasm, with cytoplasmic fluorescence intensities rising to 60% at 6 hpi and 77% at 12 hpi (Fig. 4A and B). In contrast, HSV-1 infection does not induce this translocation or significant changes in fluorescence (Fig. 4C and D), consistent with the absence of DDX46 cleavage. To investigate the relationship between DDX46 cleavage and changes in cellular localization, two truncated DDX46 mutants (1–225, 227–1,032 aa) were constructed based on the cleavage site (Fig. 4E). WT, D226A, 1–225 and 227–1,032 DDX46 were transfected into cells and subsequently infected with VSV. In the absence of infection, WT, D226A, and 1–225 DDX46 exhibited nuclear localization, while 227–1,032 DDX46 localized to both the nucleus and cytoplasm. At 12 hpi, WT, 1–225, and 227–1,032 DDX46 showed varying levels of cytoplasmic distribution, while D226A-DDX46 remained predominantly nuclear (Fig. 4F). Cytoplasmic fluorescence intensities for WT, 1–225, and 227–1,032-DDX46 were 84%, 47%, and 63%, respectively, compared to just 10% for D226A-DDX46 (Fig. 4G). These findings suggest that DDX46 cleavage promotes its translocation from the nucleus to the cytoplasm, a conclusion further supported by nucleocytoplasmic separation assays. In uninfected cells, both WT and D226A-DDX46 were confined to the nucleus (Fig. 4H). Upon VSV infection, WT-DDX46 partially translocated to the cytoplasm, while D226A-DDX46 remained exclusively nuclear (Fig. 4I and J). These results clearly demonstrate that virus-induced DDX46 cleavage contributes to its translocation from nucleus to cytoplasm.

### DDX46 cleavage boosts the IFN-β pathway in the early stages of viral infection

**Fig 5:**
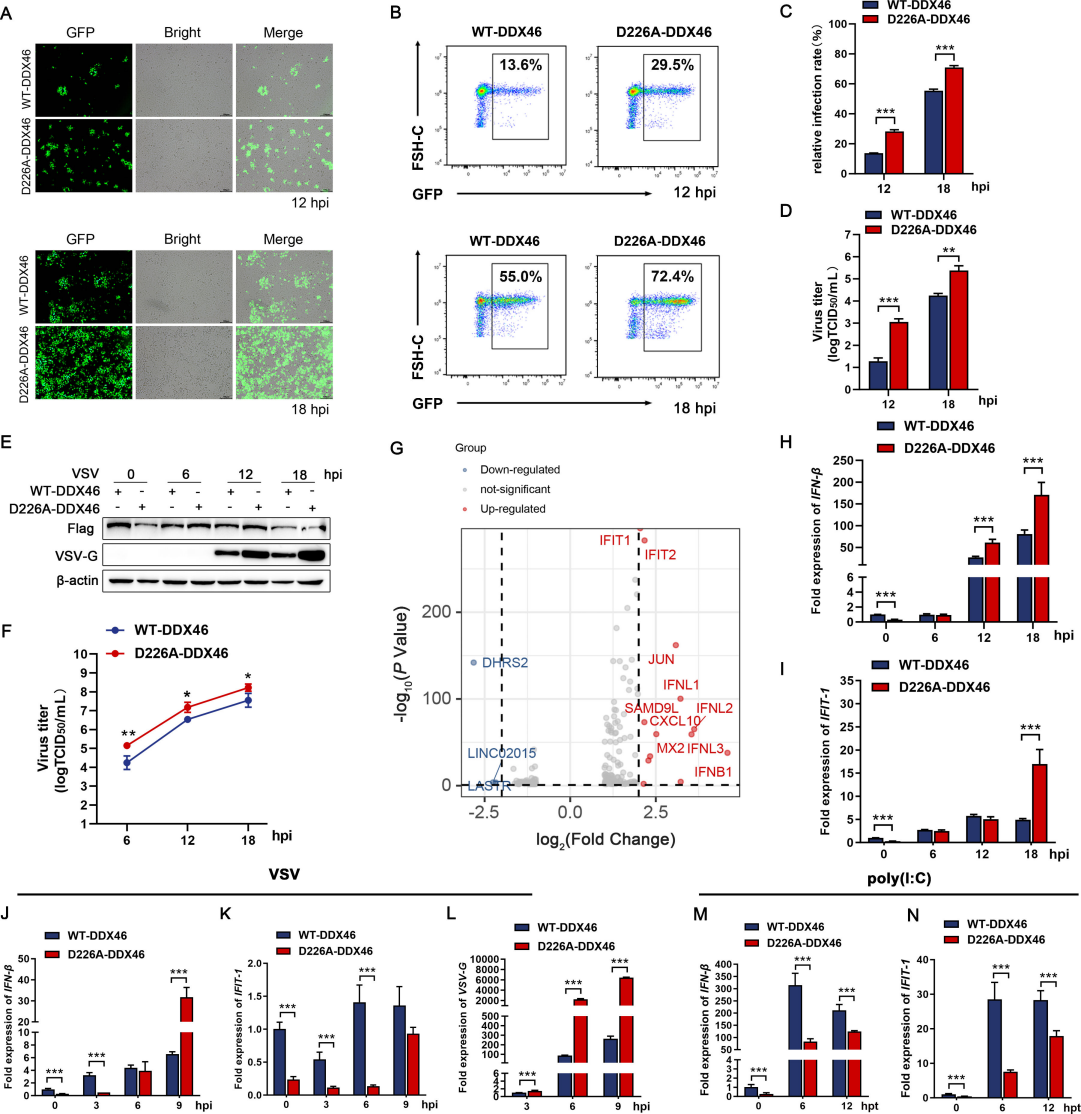
DDX46 cleavage enhances early activation of the IFN-β pathway during viral infection. (**A–D**) *DDX46*^+/-^ cells were rescued by transfection with Flag-tagged WT-DDX46 or D226A-DDX46 for 24 h, then infected with GFP-VSV (MOI = 0.1) for 12 and 18 h, followed by imaging using GFP and brightfield channels (**A**). Flow cytometry analysis with GFP on the *x*-axis and FSC-H on the *y*-axis (**B**), and quantification of relative infection rate (%) based on the flow cytometry data (**C**). Extracellular GFP-VSV titers were determined as TCID_50_ on Vero cells (**D**). (**E, F**) *DDX46*^+/-^ cells were rescued by transfection with Flag-tagged WT-DDX46 or D226A-DDX46 for 24 h, then infected with VSV (MOI = 1) for 6, 12, 18, and 24 h. Protein levels of exogenous Flag-DDX46 and VSV-G were analyzed by WB. β-Actin served as the loading control (**E**). Extracellular VSV titers were determined by TCID_50_ (**F**). (**G**) *DDX46*^+/-^ cells were rescued by transfection with Flag-tagged WT-DDX46 or D226A-DDX46 for 24 h, then infected with VSV (MOI = 1) for 12 h. Cells were harvested and subjected to transcriptome analysis, presented as a volcano plot. The *x*-axis shows log_2_(fold change) and the *y*-axis shows −log_10_(*P*-value). Red dots indicate significantly upregulated genes, blue dots indicate significantly down-regulated genes, and gray dots are non-significant. Vertical dashed lines denote fold-change cutoffs, and the horizontal dashed line marks the significance threshold. (**H and I**) Virus infection experiments were performed as in (**E**). The intracellular mRNA levels of IFN-β (**H**) and IFIT-1 (**I**) were analyzed using qRT-PCR. (**J–L**) Virus infection experiments were performed as in panel E, except the cell harvest times were changed to 0, 3, 6, and 9 hpi. The intracellular mRNA levels of IFN-β (**J**), IFIT-1 (**K**), and VSV-G (**L**) were analyzed using qRT-PCR. (**M and N**) *DDX46*^+/-^ cells were rescued by transfection with Flag-tagged WT-DDX46 or D226A-DDX46 for 24 h, then transfected with poly(I:C) (4μg/mL). The cells were lysed at 0, 6, and 12 hpt. The intracellular mRNA levels of IFN-β (**M**), IFIT-1 (**N**) were analyzed using qRT-PCR. Data are presented as means from three independent experiments. *P < 0.05, **P < 0.01, ***P < 0.001.

Since DDX46 promotes NDV and VSV replication while acting as a negative regulator of innate immunity (Fig. 1), and its cleavage facilitates translocation from the nucleus to the cytoplasm (Fig. 4), we next investigated the impact of DDX46 cleavage on innate immunity regulation and viral replication. *DDX46*^+/-^ cells were transiently transfected with WT and D226A DDX46 plasmids, followed by VSV infection. Microscopy (Fig. 5A) and flow cytometry (Fig. 5B and C) showed a significant increase in VSV-GFP replication in cells transfected with D226A DDX46 compared with WT DDX46, consistent with elevated extracellular virus titers (Fig. 5D). In time-course assays, D226A DDX46-transfected cells also exhibited higher viral protein levels and increased virus titers at 12 and 18 hpi relative to WT DDX46 (Fig. 5E and F). High-throughput transcriptome sequencing was performed to explore the function of DDX46 cleavage during virus infection (12 hpi). To our surprise, compared to WT-DDX46, the D226A-DDX46 transfected group exhibited a significant upregulation of innate immunity genes, including IFNB1 and several ISGs, such as IFIT1, IFIT2, and MX2 (Fig. 5G). These findings were further validated by measuring the mRNA levels of IFN-β and IFIT1, which showed significantly higher expression in the D226A-DDX46 group compared to the WT-DDX46 group at 12 and 18 hpi (Fig. 5H and I). Since VSV is capable of strongly inducing IFN expression, the positive correlation between viral replication levels and IFN pathway activation led us to speculate that the increased replication of the virus in the later stages of infection may contribute to the elevation of IFN and downstream ISGs. Given that DDX46 acts as a negative regulator of the IFN signaling pathway (24), we examined IFN-β and IFIT-1 expression during early VSV infection. As expected, mRNA levels of IFN-β and IFIT-1 were significantly suppressed in the D226A-DDX46 groups at 3 hpi and even at steady state compared with the WT-DDX46 groups (Fig. 5J and K). Consistently, viral RNA levels were higher in the D226A-DDX46 group, compared with the WT-DDX46 group (Fig. 5L). To avoid the indirect effects of viral replication on the results, poly(I:C) was used for validation. Consistent with expectations, the results showed that compared to WT-DDX46, the mRNA levels of IFN-β and IFIT-1 were significantly decreased in D226A-DDX46 (Fig. 5M and N). To further confirm these findings, we validated all results in *DDX46* KO cells stably expressing either WT-DDX46 or D226A-DDX46. The stable expression system provided consistent evidence supporting the observed effects of DDX46 cleavage on innate immune regulation (Fig. S3). Collectively, these findings indicate that DDX46 cleavage enhances IFN pathway activation and suppresses viral replication during early infection.

### Cleavage of DDX46 promotes nuclear export of innate immune transcripts TRAF3 and MAVS

**Fig 6:**
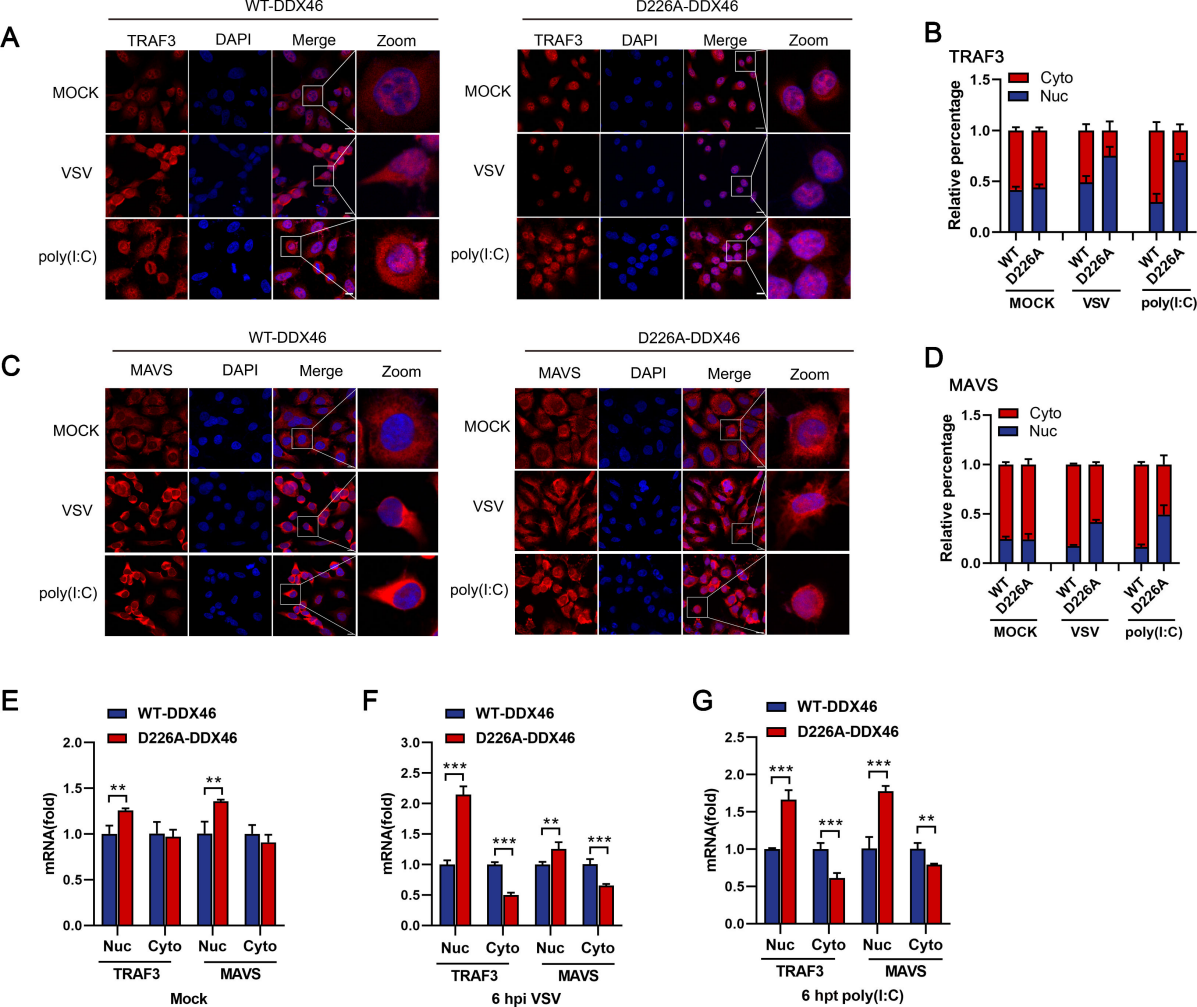
Cleavage of DDX46 promotes nuclear export of transcripts of TRAF3 and MAVS involved in innate immunity. (**A–D**) *DDX46*^+/-^ cells were rescued by transfection with Flag-tagged WT-DDX46 or D226A-DDX46 for 24 h, then mock-infected, infected with VSV (MOI = 1), or treated with poly(I:C) for 6 h. Cells were fixed and subjected to FISH analysis using probes for TRAF3 (**A and B**) and MAVS (**C and D**). Nuclei were counterstained with DAPI. Quantification of the relative percentages of cells with nuclear or cytoplasmic TRAF3 (**B**) or MAVS (**D**) mRNA signal after VSV infection. Six randomly selected fields were analyzed using ImageJ. (E to G) *DDX46*^+/-^ cells were rescued by transfection with Flag-tagged WT-DDX46 or D226A-DDX46 for 24 h, then mock-infected (**E**), infected with VSV at an MOI of 1 (**F**), or treated with poly(I:C) (**G**) for 6 h. Cells were harvested, and nuclear and cytoplasmic fractions were prepared using a nucleocytoplasmic isolation kit. The nuclear and cytoplasmic mRNA levels of TRAF3 and MAVS were analyzed using qRT-PCR. Data are presented as means from three independent experiments. **P < 0.01, ***P < 0.001.

DDX46 was reported to bind innate immune transcripts, retaining them in the nucleus and suppressing IFN production (24). Since DDX46 is cleaved and translocated from the nucleus to the cytoplasm upon viral infection, we investigated whether its cleavage disrupts its ability to trap innate immune transcripts. Consistent with previous reports, *DDX46* KO led to an increased cytoplasmic distribution of TRAF3 and MAVS mRNA under steady-state conditions (Fig. S4A through D). RNA nucleocytoplasmic isolation followed by real-time PCR analysis further confirmed the increased cytoplasmic accumulation of TRAF3 and MAVS mRNA in *DDX46*^+/-^ cells, compared with WT cells (Fig. S4E). Consistently, VSV infection and poly(I:C) treatment promote the translocation of TRAF3 and MAVS mRNA from the nucleus to the cytoplasm in cells transfected with WT-DDX46. In contrast, cells transfected with D226A-DDX46 show greater retention of TRAF3 and MAVS mRNA in the nucleus (Fig. 6A and C). Quantitative analysis shows that the nuclear fluorescence intensity proportion of TRAF3 mRNA significantly increases from 46% to 85% after VSV infection or from 28% to 77% following poly(I:C) treatment in D226A-DDX46-transfected cells, compared with a smaller increase from 38% to 44% in WT-DDX46-transfected cells. Similar trends were observed for MAVS mRNA (Fig. 6B and D). RNA nucleocytoplasmic isolation also revealed enhanced cytoplasmic accumulation of TRAF3 and MAVS mRNA in D226A-DDX46-transfected cells following VSV infection and poly(I:C) treatment, compared to WT-DDX46-transfected cells (Fig. 6E through G). These findings clearly demonstrate that the cleavage of DDX46 is crucial for its role in regulating the nucleocytoplasmic distribution of innate immune transcripts during antiviral responses.

## DISCUSSION

**Fig 7:**
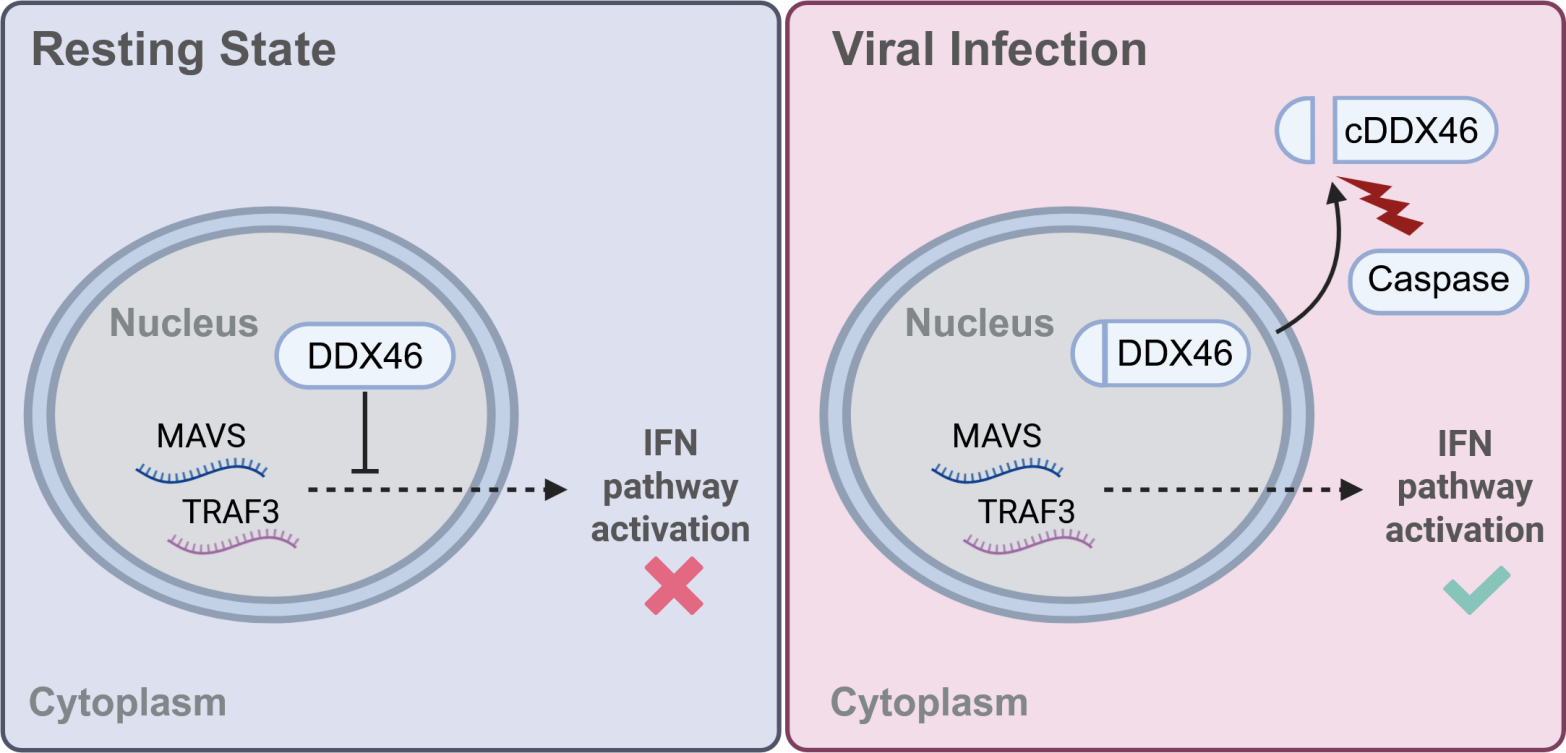
Physiological state and viral infection conditions: the regulatory role of DDX46 in the host innate immune pathway. This describes how DDX46 is cleaved and its effects during RNA virus infection. Under physiological conditions, DDX46 is located in the nucleus, where it suppresses the nuclear export of transcripts for innate immune molecules like MAVS and TRAF3, thereby preventing excessive activation of the IFN pathway. However, upon RNA virus stimulation, the host induces the cleavage of DDX46 via caspase, promoting its nuclear export. This, in turn, facilitates the nuclear export of MAVS and TRAF3 transcripts, thereby enhancing the IFN pathway.

Recent research into the functions and structures of RNA-binding proteins, particularly the DEAD/H-box RNA helicase family, has revealed their dual roles in RNA synthesis and metabolism, as well as in the regulation of innate immunity (28–30). Although a complex interplay exists between viruses and hosts involving the DEAD/H-box RNA helicase family, the specific helicases essential for viral replication and antiviral innate immunity remain unclear. In this study, we utilized an RBP knockout sub-library to identify key members of the DEAD/H-box RNA helicases that influence host cell survival upon viral challenge. Notably, helicases such as DDX24 and DDX5 have previously been recognized as critical regulators of innate immune responses and the viral life cycle, further supporting the validity of our screening results (31–34). In this study, we identified DDX46 as a key host factor that negatively regulates antiviral innate immunity. Importantly, our findings reveal that RNA viruses induce the cleavage of DDX46, leading to its translocation from the nucleus to the cytoplasm. This cleavage event “releases” the innate immune transcripts, allowing them to exit the nucleus and activate downstream innate immune pathways, thereby promoting an effective antiviral response (Fig. 7).

The DEAD/H-box helicase family serves as a key regulator of host immune responses against RNA viruses, playing a central role in antiviral defense mechanisms (35). Positioned at the frontline of the ongoing “arms race” between viruses and the host immune system, RNA helicases are fiercely contested targets, and both viruses and hosts have evolved diverse strategies to modulate their activity (36, 37). Compared with transcriptional regulation, post-translational control offers greater flexibility and efficiency, making it a principal mechanism for modulating protein function (38). For example, RIG-I signaling can be enhanced by host-mediated ubiquitination and deacetylation following viral infection, while various viruses suppress RIG-I activity through degradation or dephosphorylation (39–41). By comparison, the cleavage of RNA helicases during virus infection has been relatively understudied. During Dengue virus (DENV) infection, the viral NS2B/3 protease cleaves DDX21, suppressing innate immunity and promoting DENV replication (42). Our previous study also showed that viral infection induces DDX21 cleavage, disrupting the DDX1-DDX21-DHX36 complex and suppressing the host innate immune response (19). In contrast to DDX21, which promotes interferon production, DDX46 functions as a host factor that suppresses interferon induction and facilitates viral replication, a role that is inherently disadvantageous for the host (24). Our study demonstrates that the cleavage of DDX46 represents a flexible regulatory mechanism by which the host can relieve this suppression and enhance antiviral responses. In addition to DDX46, other RNA helicases, such as DDX24 and DDX5, have also been shown to negatively regulate innate immune signaling (31, 43). However, whether these RNA helicases undergo post-translational modifications that alter or abolish their inhibitory function on innate immunity remains to be further investigated. This underscores not only the functional diversity of RNA helicases in innate immunity, but also the dynamic evolutionary interplay between viruses and their hosts, potentially offering novel targets for future antiviral therapies.

The biological functions of RBPs are closely linked to their cellular localization. Under conditions such as cellular stress or pathogen infection, the localization of certain RBPs can shift, allowing them to perform new or enhanced functions that help the cell adapt to environmental changes (19, 44). For example, following Sindbis virus (SINV) infection, the host selectively translocates specific ribonucleoproteins (RNPs), notably U2 small nuclear RNPs (snRNPs), to the cytoplasm, which significantly suppresses SINV gene expression (45). Additionally, in resting cells, polypyrimidine tract-binding protein (PTB) is mainly nuclear. Upon Japanese encephalitis virus (JEV) infection, PTB relocates to the cytoplasm, where it interferes with the binding of JEV 3NCR (-) RNA to the viral RNA polymerase (NS5), thereby inhibiting viral replication (46). In this study, we found that DDX46 is strictly localized to the nucleus under physiological conditions. Due to antibody non-specificity, endogenous DDX46 could not be reliably visualized by IF; thus, we relied on Flag-tagged DDX46 to assess localization, which showed predominant nuclear distribution in resting cells, consistent with its functional involvement in U2 snRNP assembly (20). Upon infection with the RNA virus, DDX46 was cleaved followed by translocating from the nucleus to the cytoplasm. These findings further underscore the dynamic subcellular localization of RBPs as a crucial regulatory mechanism during viral infection. In contrast to RNA viruses, infection with the DNA viruses does not alter the subcellular localization of DDX46. These differences may reflect distinct signaling pathways activated by RNA and DNA viruses (47–53). Previous studies in our lab have shown that NDV can target MAVS for degradation via the E3 ubiquitin ligase RNF5 (54). Moreover, RNA viruses, such as Hepatitis C virus, rotavirus, and Seneca Valley virus, can also target MAVS for degradation or cleavage through different mechanisms, thereby suppressing the host’s innate immune response (55, 56). Notably, the absence of DDX46 can restore MAVS protein levels (24). Therefore, we hypothesize that RNA viruses, but not DNA viruses, induce DDX46 cleavage, potentially as a compensatory response by the host cell to the MAVS depletion triggered by RNA virus infection. Consistent with reports that DDX46-ALKBH5-mediated m6A demethylation promotes nuclear retention and dampens IFN responses (24), our data indicate that DDX46 cleavage enhances its nuclear export and associates with shifts in the nucleo-cytoplasmic distribution of antiviral mRNAs.

Various RBPs, localized in the nucleus, play important roles in maintaining genome stability (19, 45). This may explain why several DEAD/H-box RNA helicases are essential for mouse development, as knockout of *Ddx* genes such as *Ddx24*, *Ddx5*, and *Ddx17* results in early embryonic lethality (31, 57). Previous reports showed that *Ddx46*^-/-^ mice die *in utero* (24). Similarly, in our study, only heterozygous *DDX46* knockout cells could be obtained. These results collectively demonstrate that these RNA helicases may not be suitable as drug targets to regulate immune responses or inhibit viral infection due to their essential cellular functions. However, considering evidence from our work and others’ studies showing that specific amino acid residues in RNA helicases are critical for antiviral innate immunity (19, 58), targeting these sites may provide an alternative strategy to specifically regulate immune responses without disrupting their primary roles in maintaining cell stability. More detailed studies are needed to elucidate the underlying mechanisms of post-translational modifications in DEAD/H-box RNA helicases, which could inform the rational design of targeted therapeutics.

Our studies showed that DDX46 cleavage promotes VSV replication and, unexpectedly, is accompanied by enhanced activation of the IFN pathway at the late stage of infection. This finding is in agreement with the well-established ability of VSV to strongly stimulate IFN signaling and the expression of genes associated with type I IFN pathways (59). By assessing IFN signaling at the early stage of VSV infection and following poly(I:C) stimulation, we demonstrated that DDX46 cleavage indeed promotes IFN production while simultaneously inhibiting viral mRNA levels. Our findings indicate that DDX46 cleavage regulates innate immunity during the early phase of infection, representing a rapid response mechanism similar to other RNA helicases. For example, endogenous RIG-I recognizes viral RNA and promptly initiates antiviral innate immune responses (60). Together, these findings underscore the contribution of DDX46 and other RNA helicases to rapid innate immune defense against RNA viruses and to shaping early antiviral response kinetics.

Notably, the magnitude of the phenotypes observed upon DDX46 perturbation is modest in several assays, and under some conditions, viruses reach similar endpoint titers. These observations highlight several limitations of our study. First, DDX46 is unlikely to represent a major rate-limiting determinant of VSV or NDV replication in the experimental systems used here, and its effects on IFN signaling are best viewed as fine-tuning changes in pathway dynamics. Second, because complete loss of DDX46 is not tolerated in our system, we were restricted to siRNA knockdown and heterozygous knockout models, which likely retain residual DDX46 activity and may underestimate the full contribution of DDX46 to antiviral signaling. Third, our experiments were performed primarily in immortalized cell lines and *in vitro* infection models using a limited set of RNA viruses, and therefore may not fully capture the complexity of *in vivo* antiviral responses or the range of pathogens regulated by DDX46. At the same time, these modest effect sizes are consistent with the redundancy and robustness of the type I IFN network, where incremental changes at individual nodes can still influence early kinetics and downstream amplification through paracrine IFN signaling. Finally, at the mechanistic level, although our data support a model in which DDX46 cleavage influences the nucleo-cytoplasmic distribution of MAVS and TRAF3 mRNAs, the present experiments do not directly establish that altered mRNA export is necessary and sufficient to explain the observed effects on IFN signaling, and this mechanism will require further investigation.

In summary, our study provides new insights into the multifaceted roles of DDX46 in the regulation of antiviral innate immunity. While previous studies showed that DDX46 retains demethylated innate immune transcripts in the nucleus to suppress IFN production (24), we demonstrate that RNA virus infection triggers DDX46 cleavage and its translocation to the cytoplasm, thereby releasing these transcripts and enhancing antiviral responses. In this context, our findings are consistent with a role for DDX46 in modulating antiviral signaling dynamics within the broader innate immune network. These findings emphasize the functional diversity of RNA helicases and the importance of post-translational modification and subcellular localization in immune regulation, suggesting potential directions for antiviral therapeutic development.

## Data Availability

All data of this study are available from the corresponding author upon request, without undue reservation. All relevant data are within the article and its supplemental material. The raw data can be found at the following link. https://zenodo.org/records/15827173?preview=1&token=eyJhbGciOiJIUzUxMiJ9.eyJpZCI6IjAwZGU1N2UzLTI3NWQtNGE3YS04NDAyLWVmYmE2OGU5MzVjYSIsImRhdGEiOnt9LCJyYW5kb20iOiJmZGZjYWNlMDI5ZTkwYjZkYWQzOWU4ZmE4ZmQ4NzQ1NyJ9.4x_bL2cTIofpbvp5fAaXiMMkGRFhov_9kpg-CxktNpLrKuJvG_6qjB1O_MM8vVdwdgUL-9kTsrVKGoVNLXQs-w.

## SUPPLEMENTAL MATERIAL

Fig. S1 mbio.03519-25-s0001.tif DDX46 negatively regulates the IFN-β signaling pathway.

Fig. S2 mbio.03519-25-s0002.tif The cleavage of DDX46 by virus infection in various cell types.

Fig. S3 mbio.03519-25-s0003.tif DDX46 cleavage promotes IFN-β pathway early in viral infection.

Fig. S4 mbio.03519-25-s0004.tif DDX46 regulates the nuclear export of TRAF3 and MAVS transcripts.

Supplemental legends mbio.03519-25-s0005.docx Legends for all supplemental figures and tables.

Table S1 mbio.03519-25-s0006.docx Primers used in this study.

Table S2 mbio.03519-25-s0007.xlsx Enriched genes identified in the RBP knockout library screen.

ASM does not own the copyrights to Supplemental Material that may be linked to, or accessed through, an article. The authors have granted ASM a non-exclusive, world-wide license to publish the Supplemental Material files. Please contact the corresponding author directly for reuse.

## References

[1] Liu ZP, Liu S, Chen R, Huang X, Wu LY. 2017. Structure alignment-based classification of RNA-binding pockets reveals regional RNA recognition motifs on protein surfaces. BMC Bioinformatics 18:27. doi:10.1186/s12859-016-1410-1 28077065 PMC5225598

[2] Burd CG, Dreyfuss G. 1994. Conserved structures and diversity of functions of RNA-binding proteins. Science 265:615–621. doi:10.1126/science.8036511 8036511

[3] Beckmann BM, Castello A, Medenbach J. 2016. The expanding universe of ribonucleoproteins: of novel RNA-binding proteins and unconventional interactions. Pflugers Arch 468:1029–1040. doi:10.1007/s00424-016-1819-4 27165283 PMC4893068

[4] Garcia-Moreno M, Järvelin AI, Castello A. 2018. Unconventional RNA-binding proteins step into the virus-host battlefront. Wiley Interdiscip Rev RNA 9:e1498. 30091184 10.1002/wrna.1498 PMC7169762

[5] Shurong L, Bin L, Qiaoxia L. 2020. Classification and function of RNA-protein interactions. Wiley Interdiscip Rev RNA 11:e1601. 32488992 10.1002/wrna.1601

[6] Corley M, Burns MC, Yeo GW. 2020. How RNA-binding proteins interact with RNA: molecules and mechanisms. Mol Cell 78:9–29. doi:10.1016/j.molcel.2020.03.011 32243832 PMC7202378

[7] Dong X-Y, Liu W-J, Zhao M-Q, Wang J-Y, Pei J-J, Luo Y-W, Ju C-M, Chen J-D. 2013. Classical swine fever virus triggers RIG-I and MDA5-dependent signaling pathway to IRF-3 and NF-κB activation to promote secretion of interferon and inflammatory cytokines in porcine alveolar macrophages. Virol J 10:286. doi:10.1186/1743-422X-10-286 24034559 PMC3849481

[8] Loo Y-M, Fornek J, Crochet N, Bajwa G, Perwitasari O, Martinez-Sobrido L, Akira S, Gill MA, García-Sastre A, Katze MG, Gale M Jr. 2008. Distinct RIG-I and MDA5 signaling by RNA viruses in innate immunity. J Virol 82:335–345. doi:10.1128/JVI.01080-07 17942531 PMC2224404

[9] Kato H, Sato S, Yoneyama M, Yamamoto M, Uematsu S, Matsui K, Tsujimura T, Takeda K, Fujita T, Takeuchi O, Akira S. 2005. Cell type-specific involvement of RIG-I in antiviral response. Immunity 23:19–28. doi:10.1016/j.immuni.2005.04.010 16039576

[10] Eberhardt W, Badawi A, Biyanee A, Pfeilschifter J. 2016. Cytoskeleton-dependent transport as a potential target for interfering with post-transcriptional HuR mRNA regulons. Front Pharmacol 7:251. doi:10.3389/fphar.2016.00251 27582706 PMC4987335

[11] Guha A, Husain MA, Si Y, Nabors LB, Filippova N, Promer G, Smith R, King PH. 2023. RNA regulation of inflammatory responses in glia and its potential as a therapeutic target in central nervous system disorders. Glia 71:485–508. doi:10.1002/glia.24288 36380708

[12] Ouhara K, Munenaga S, Kajiya M, Takeda K, Matsuda S, Sato Y, Hamamoto Y, Iwata T, Yamasaki S, Akutagawa K, Mizuno N, Fujita T, Sugiyama E, Kurihara H. 2018. The induced RNA-binding protein, HuR, targets 3′-UTR region of IL-6 mRNA and enhances its stabilization in periodontitis. Clin Exp Immunol 192:325–336. doi:10.1111/cei.13110 29393507 PMC5980314

[13] Cao D, Bian J, Hua Z-C. 2015. Modulation of TNF-α mRNA stability by human antigen R and miR181s in sepsis-induced immunoparalysis. EMBO Mol Med 7:140–157. doi:10.15252/emmm.201404797 25535255 PMC4328645

[14] Fairman-Williams ME, Guenther U-P, Jankowsky E. 2010. SF1 and SF2 helicases: family matters. Curr Opin Struct Biol 20:313–324. doi:10.1016/j.sbi.2010.03.011 20456941 PMC2916977

[15] Michael P, Matthew E L, Emily A H. 2024. Established and emerging roles of DEAD/H-box helicases in regulating infection and immunity. Immunol Rev10.1111/imr.13426 PMC11741935 39620586

[16] Thulasi Raman SN, Liu G, Pyo HM, Cui YC, Xu F, Ayalew LE, Tikoo SK, Zhou Y. 2016. DDX3 interacts with influenza A virus NS1 and NP proteins and exerts antiviral function through regulation of stress granule formation. J Virol 90:3661–3675. doi:10.1128/JVI.03010-15 26792746 PMC4794679

[17] Niu Q, Cheng Y, Wang H, Yan Y, Sun J. 2019. Chicken DDX3X activates IFN-β via the chSTING-chIRF7-IFN-β signaling axis. Front Immunol 10. doi:10.3389/fimmu.2019.00822 PMC6478769 31057547

[18] Zhang Z, Kim T, Bao M, Facchinetti V, Jung SY, Ghaffari AA, Qin J, Cheng G, Liu Y-J. 2011. DDX1, DDX21, and DHX36 helicases form a complex with the adaptor molecule TRIF to sense dsRNA in dendritic cells. Immunity 34:866–878. doi:10.1016/j.immuni.2011.03.027 21703541 PMC3652560

[19] Wu W, Qu Y, Yu S, Wang S, Yin Y, Liu Q, Meng C, Liao Y, Ur Rehman Z, Tan L, Song C, Qiu X, Liu W, Ding C, Sun Y. 2021. Caspase-dependent cleavage of DDX21 suppresses host innate immunity. mBio 12:e0100521. doi:10.1128/mBio.01005-21 34125604 PMC8262918

[20] Yang F, Bian T, Zhan X, Chen Z, Xing Z, Larsen NA, Zhang X, Shi Y. 2023. Mechanisms of the RNA helicases DDX42 and DDX46 in human U2 snRNP assembly. Nat Commun 14. doi:10.1038/s41467-023-36489-x PMC9935549 36797247

[21] Soller M. 2006. Pre-messenger RNA processing and its regulation: a genomic perspective. Cell Mol Life Sci 63:796–819. doi:10.1007/s00018-005-5391-x 16465448 PMC11136025

[22] Li M, Ma Y, Huang P, Du A, Yang X, Zhang S, Xing C, Liu F, Cao J. 2015. Lentiviral DDX46 knockdown inhibits growth and induces apoptosis in human colorectal cancer cells. Gene 560:237–244. doi:10.1016/j.gene.2015.02.020 25680556

[23] Ma J, Gao Z, Liu X. 2021. DDX46 accelerates the proliferation of glioblastoma by activating the MAPK-p38 signaling. J BUON 26:2084–2089. 34761620

[24] Zheng Q, Hou J, Zhou Y, Li Z, Cao X. 2017. The RNA helicase DDX46 inhibits innate immunity by entrapping m6A-demethylated antiviral transcripts in the nucleus. Nat Immunol 18:1094–1103. doi:10.1038/ni.3830 28846086

[25] Sun Y, Tang L, Kan X, Tan L, Song C, Qiu X, Liao Y, Nair V, Ding C, Liu X, Sun Y. 2024. Oncolytic Newcastle disease virus induced degradation of YAP through E3 ubiquitin ligase PRKN to exacerbate ferroptosis in tumor cells. J Virol 98. doi:10.1128/jvi.01897-23 PMC10949840 38411946

[26] Cao P, Luo W-W, Li C, Tong Z, Zheng Z-Q, Zhou L, Xiong Y, Li S. 2019 The heterogeneous nuclear ribonucleoprotein hnRNPM inhibits RNA virus-triggered innate immunity by antagonizing RNA sensing of RIG-I-like receptors. PLoS Pathog 15:e1007983. doi:10.1371/journal.ppat.1007983 31433824 PMC6703689

[27] Wang P, Meng W, Han S-C, Li C-C, Wang X-J, Wang X-J. 2016. The nucleolar protein GLTSCR2 is required for efficient viral replication. Sci Rep 6. doi:10.1038/srep36226 PMC5099953 27824081

[28] Kok K-H, Jin D-Y. 2013. Balance of power in host-virus arms races. Cell Host Microbe 14:5–6. doi:10.1016/j.chom.2013.07.004 23870307

[29] Heaton SM, Gorry PR, Borg NA. 2023. DExD/H-box helicases in HIV-1 replication and their inhibition. Trends Microbiol 31:393–404. doi:10.1016/j.tim.2022.11.001 36463019

[30] Castello A, Álvarez L, Kamel W, Iselin L, Hennig J. 2024. Exploring the expanding universe of host-virus interactions mediated by viral RNA. Mol Cell 84:3706–3721. doi:10.1016/j.molcel.2024.08.027 39366356

[31] Ma Z, Moore R, Xu X, Barber GN. 2013. DDX24 negatively regulates cytosolic RNA-mediated innate Immune Signaling. PLoS Pathog 9:e1003721. doi:10.1371/journal.ppat.1003721 24204270 PMC3814876

[32] Khatun M, Zhang J, Ray R, Ray RB. 2021. Hepatitis C virus evades interferon signaling by suppressing long noncoding RNA linc-pint involving C/EBP-β. J Virol 95:e0095221. doi:10.1128/JVI.00952-21 34160260 PMC8354323

[33] Xu J, Cai Y, Ma Z, Jiang B, Liu W, Cheng J, Guo N, Wang Z, Sealy JE, Song C, Wang X, Li Y. n.d. The RNA helicase DDX5 promotes viral infection via regulating N6-methyladenosine levels on the DHX58 and NFκB transcripts to dampen antiviral innate immunity. PLoS Pathog 17:e1009530. doi:10.1371/journal.ppat.1009530 PMC8081163 33909701

[34] Zhang Y, Cen J, Yuan G, Jia Z, Chen K, Gao W, Chen J, Adamek M, Jia Z, Zou J. 2023. DDX5 inhibits type I IFN production by promoting degradation of TBK1 and disrupting formation of TBK1 − TRAF3 complex. Cell Mol Life Sci 80:212. doi:10.1007/s00018-023-04860-2 37462751 PMC11073175

[35] Taschuk F, Cherry S. 2020. DEAD-box helicases: sensors, regulators, and effectors for antiviral defense. Viruses 12:181. doi:10.3390/v12020181 32033386 PMC7077277

[36] Baldaccini M, Pfeffer S. n.d. Untangling the roles of RNA helicases in antiviral innate immunity. PLoS Pathog 17:e1010072. doi:10.1371/journal.ppat.1010072 PMC8659333 34882751

[37] Tapescu I, Cherry S. 2024. DDX RNA helicases: key players in cellular homeostasis and innate antiviral immunity. J Virol 98. doi:10.1128/jvi.00040-24 PMC11494928 39212449

[38] Jin J, Zhang R, Li J, Gao F, Liao Z, Yu Y, Wang Y, Bucci D, Xiao M, Ma R, Ma Q, Gao S, Lio J, Novais F, Huang S-C, Zhu J, Ghoneim H, Wen H, Li Z, Sun N, Xin G. 2025. The NAE1-mediated neddylation operates as an essential post-translational modification checkpoint for effector CD8^+^ T cells. Proc Natl Acad Sci USA 122:e2424061122. doi:10.1073/pnas.2424061122 40030035 PMC11912420

[39] Li Z, Xiao W, Yang Z, Guo J, Zhou J, Xiao S, Fang P, Fang L. 2024. Cleavage of HDAC6 to dampen its antiviral activity by nsp5 is a common strategy of swine enteric coronaviruses. J Virol 98:e0181423. doi:10.1128/jvi.01814-23 38289103 PMC10878235

[40] Liu HM, Jiang F, Loo YM, Hsu S, Hsiang T-Y, Marcotrigiano J, Gale M Jr. 2016. Regulation of retinoic acid inducible gene-I (RIG-I) activation by the histone deacetylase 6. EBioMedicine 9:195–206. doi:10.1016/j.ebiom.2016.06.015 27372014 PMC4972567

[41] Gack MU, Shin YC, Joo CH, Urano T, Liang C, Sun L, Takeuchi O, Akira S, Chen Z, Inoue S, Jung JU. 2007. TRIM25 RING-finger E3 ubiquitin ligase is essential for RIG-I-mediated antiviral activity. Nature 446:916–920. doi:10.1038/nature05732 17392790

[42] Dong Y, Ye W, Yang J, Han P, Wang Y, Ye C, Weng D, Zhang F, Xu Z, Lei Y. 2016. DDX21 translocates from nucleus to cytoplasm and stimulates the innate immune response due to dengue virus infection. Biochem Biophys Res Commun 473:648–653. doi:10.1016/j.bbrc.2016.03.120 27033607

[43] Zhang Y, Cen J, Yuan G, Jia Z, Chen K, Gao W, Chen J, Adamek M, Jia Z, Zou J. 2023. DDX5 inhibits type I IFN production by promoting degradation of TBK1 and disrupting formation of TBK1 − TRAF3 complex. Cell Mol Life Sci 80. doi:10.1007/s00018-023-04860-2 PMC11073175 37462751

[44] Song Y, Guo Y, Li X, Sun R, Zhu M, Shi J, Tan Z, Zhang L, Huang J. 2021 RBM39 alters phosphorylation of c-jun and binds to viral RNA to promote PRRSV proliferation. Front Immunol 12. doi:10.3389/fimmu.2021.664417 PMC8165236 34079549

[45] Kamel W, Ruscica V, Embarc-Buh A, de Laurent ZR, Garcia-Moreno M, Demyanenko Y, Orton RJ, Noerenberg M, Madhusudhan M, Iselin L, Järvelin AI, Hannan M, Kitano E, Moore S, Merits A, Davis I, Mohammed S, Castello A. 2024. Alphavirus infection triggers selective cytoplasmic translocation of nuclear RBPs with moonlighting antiviral roles. Mol Cell 84:4896–4911. doi:10.1016/j.molcel.2024.11.015 39642884

[46] Bhullar D, Jalodia R, Kalia M, Vrati S. 2014. Cytoplasmic translocation of polypyrimidine tract-binding protein and its binding to viral RNA during Japanese encephalitis virus infection inhibits virus replication. PLoS One 9:e114931. doi:10.1371/journal.pone.0114931 25545659 PMC4278868

[47] Singh RS, Vidhyasagar V, Yang S, Arna AB, Yadav M, Aggarwal A, Aguilera AN, Shinriki S, Bhanumathy KK, Pandey K, Xu A, Rapin N, Bosch M, DeCoteau J, Xiang J, Vizeacoumar FJ, Zhou Y, Misra V, Matsui H, Ross SR, Wu Y. 2022. DDX41 is required for cGAS-STING activation against DNA virus infection. Cell Rep 39:110856. doi:10.1016/j.celrep.2022.110856 35613581 PMC9205463

[48] Wang Y, Ning X, Gao P, Wu S, Sha M, Lv M, Zhou X, Gao J, Fang R, Meng G, Su X, Jiang Z. 2017. Inflammasome activation triggers caspase-1-mediated cleavage of cGAS to regulate responses to DNA Virus Infection. Immunity 46:393–404. doi:10.1016/j.immuni.2017.02.011 28314590

[49] Wu Y, Song K, Hao W, Li J, Wang L, Li S. 2022. Nuclear soluble cGAS senses double-stranded DNA virus infection. Commun Biol 5:433. doi:10.1038/s42003-022-03400-1 35538147 PMC9090744

[50] Yoneyama M, Kato H, Fujita T. 2024. Physiological functions of RIG-I-like receptors. Immunity 57:731–751. doi:10.1016/j.immuni.2024.03.003 38599168

[51] Thorne LG, Reuschl AK, Zuliani-Alvarez L, Whelan MVX, Turner J, Noursadeghi M, Jolly C, Towers GJ. 2021. SARS-CoV-2 sensing by RIG-I and MDA5 links epithelial infection to macrophage inflammation. EMBO J 40:e107826. doi:10.15252/embj.2021107826 34101213 PMC8209947

[52] Rehwinkel J, Tan CP, Goubau D, Schulz O, Pichlmair A, Bier K, Robb N, Vreede F, Barclay W, Fodor E, Reis e Sousa C. 2010. RIG-I detects viral genomic RNA during negative-strand RNA virus infection. Cell 140:397–408. doi:10.1016/j.cell.2010.01.020 20144762

[53] Yang W, Ru Y, Ren J, Bai J, Wei J, Fu S, Liu X, Li D, Zheng H. 2019. G3BP1 inhibits RNA virus replication by positively regulating RIG-I-mediated cellular antiviral response. Cell Death Dis 10:946. doi:10.1038/s41419-019-2178-9 31827077 PMC6906297

[54] Sun Y, Zheng H, Yu S, Ding Y, Wu W, Mao X, Liao Y, Meng C, Ur Rehman Z, Tan L, Song C, Qiu X, Wu F, Ding C. 2019. Newcastle disease virus V protein degrades mitochondrial antiviral signaling protein to inhibit host type I interferon production via E3 ubiquitin ligase RNF5. J Virol 93. doi:10.1128/JVI.00322-19 PMC6714796 31270229

[55] Qin Y, Xue B, Liu C, Wang X, Tian R, Xie Q, Guo M, Li G, Yang D, Zhu H. 2017. NLRX1 mediates MAVS degradation to attenuate the hepatitis c virus-induced innate immune response through PCBP2. J Virol 91. doi:10.1128/JVI.01264-17 PMC5686720 28956771

[56] Qian S, Fan W, Liu T, Wu M, Zhang H, Cui X, Zhou Y, Hu J, Wei S, Chen H, Li X, Qian P. 2017. Seneca valley virus suppresses host type i interferon production by targeting adaptor proteins MAVS, TRIF, and TANK for Cleavage. J Virol 91. doi:10.1128/JVI.00823-17 PMC5533933 28566380

[57] Ralf Janknecht. 2010. Multi-talented DEAD-box proteins and potential tumor promoters: p68 RNA helicase (DDX5) and its paralog, p72 RNA helicase (DDX17). Am J Transl Res 2:223–234. 20589163 PMC2892403

[58] Wang W, Jia M, Zhao C, Yu Z, Song H, Qin Y, Zhao W. 2021. RNF39 mediates K48-linked ubiquitination of DDX3X and inhibits RLR-dependent antiviral immunity. Sci Adv 7. doi:10.1126/sciadv.abe5877 PMC7935364 33674311

[59] Panda D, Dinh PX, Beura LK, Pattnaik AK. 2010. Induction of interferon and interferon signaling pathways by replication of defective interfering particle RNA in cells constitutively expressing vesicular stomatitis virus replication proteins. J Virol 84:4826–4831. doi:10.1128/JVI.02701-09 20181705 PMC2863789

[60] Thoresen DT, Galls D, Götte B, Wang W, Pyle AM. 2023. A rapid RIG-I signaling relay mediates efficient antiviral response. Mol Cell 83:90–104. doi:10.1016/j.molcel.2022.11.018 36521492 PMC9825657

