# Supplementary figures and images for: Caspase-mediated DDX46 cleavage unchains antiviral immunity
Source: mBio. 2026 Mar 19;17(4):e03519-25. doi: 10.1128/mbio.03519-25 (PMC13059769; doi:10.1128/mbio.03519-25)

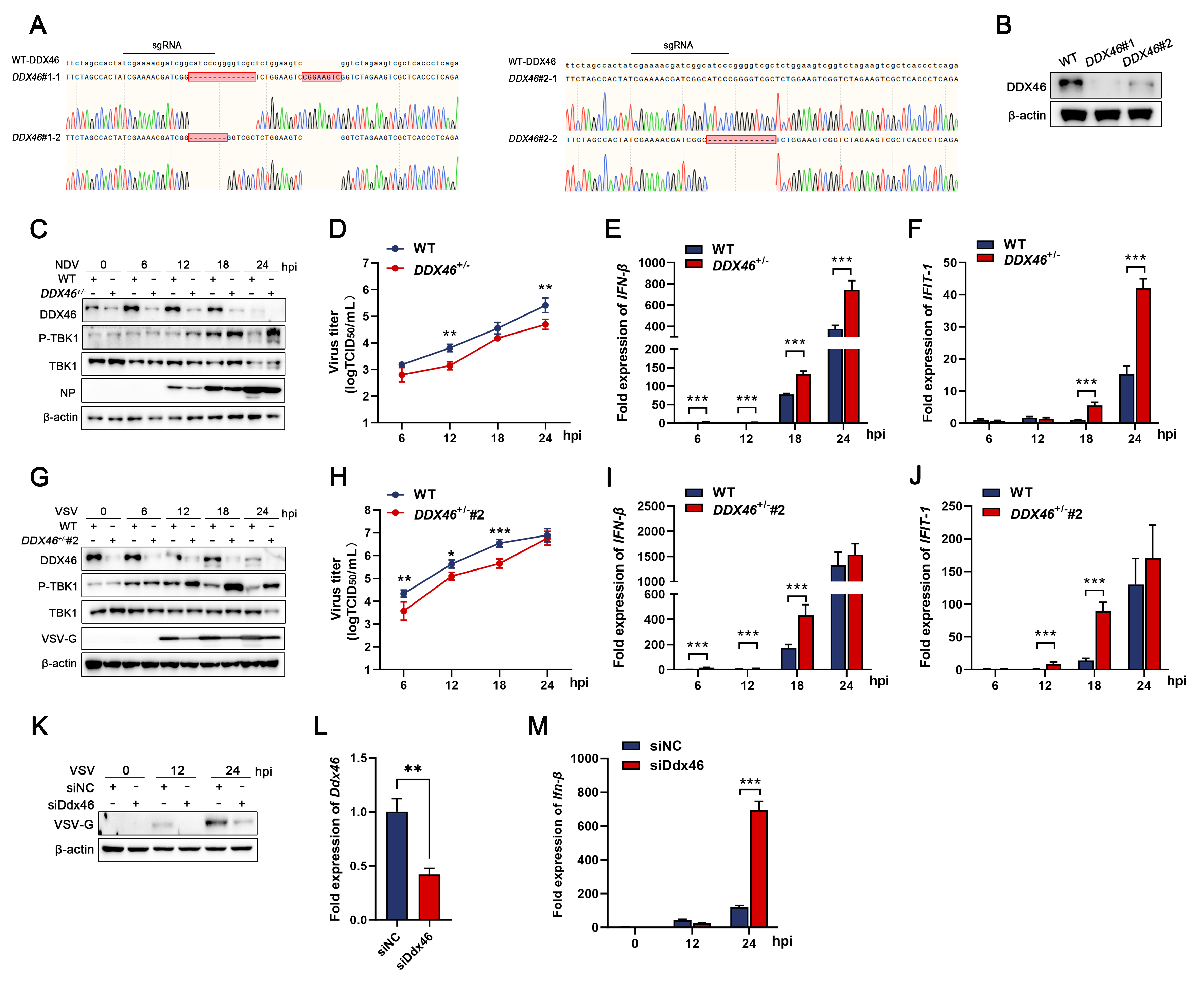

Supplement: Fig. S1 — DDX46 negatively regulates the IFN-β signaling pathway. [file mbio.03519-25-s0001.tif]

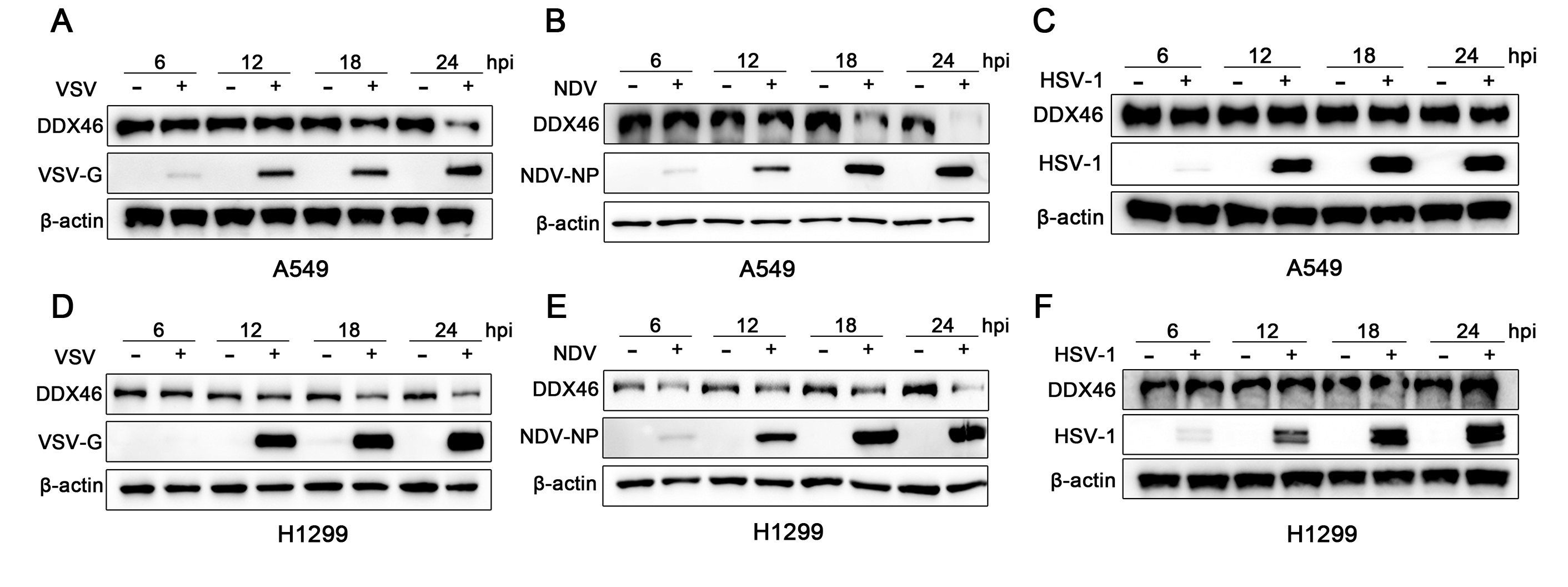

Supplement: Fig. S2 — The cleavage of DDX46 by virus infection in various cell types. [file mbio.03519-25-s0002.tif]

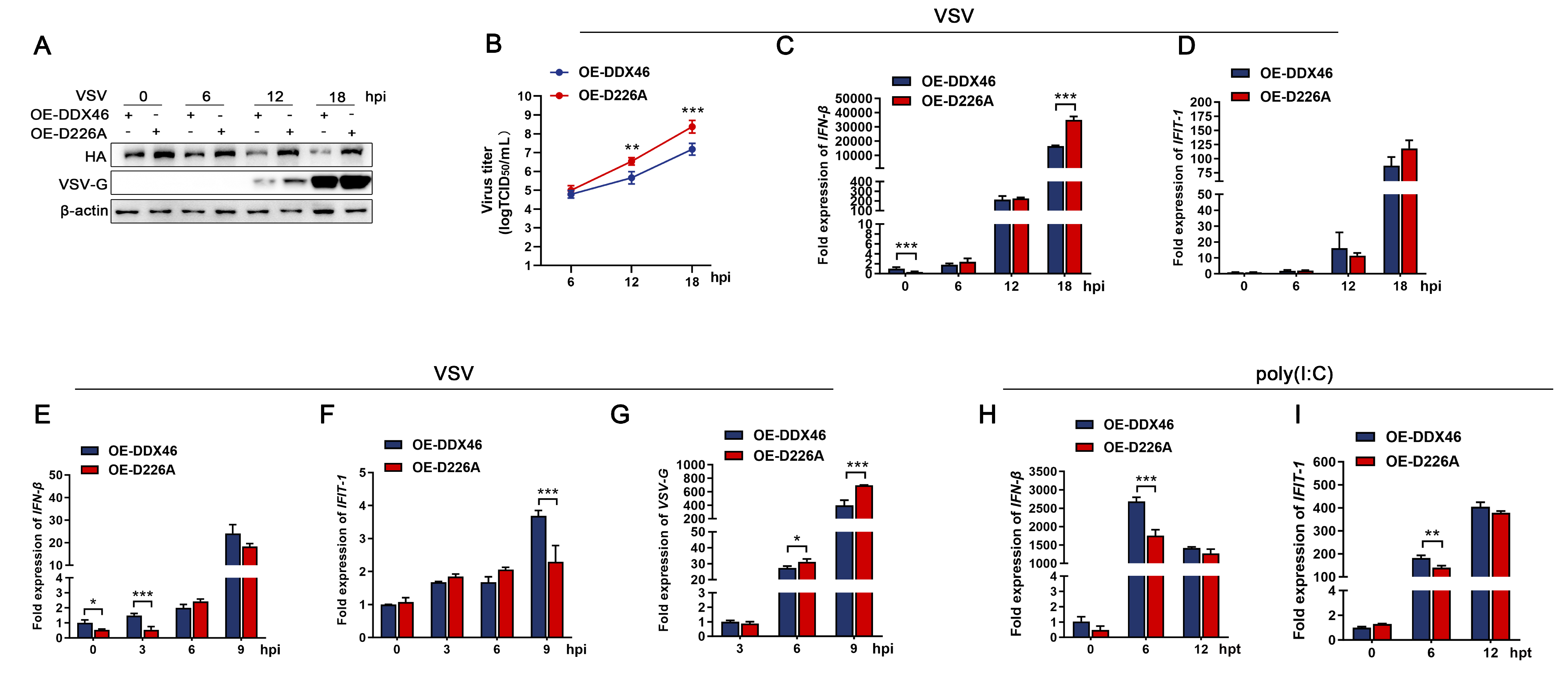

Supplement: Fig. S3 — DDX46 cleavage promotes IFN-β pathway early in viral infection. [file mbio.03519-25-s0003.tif]

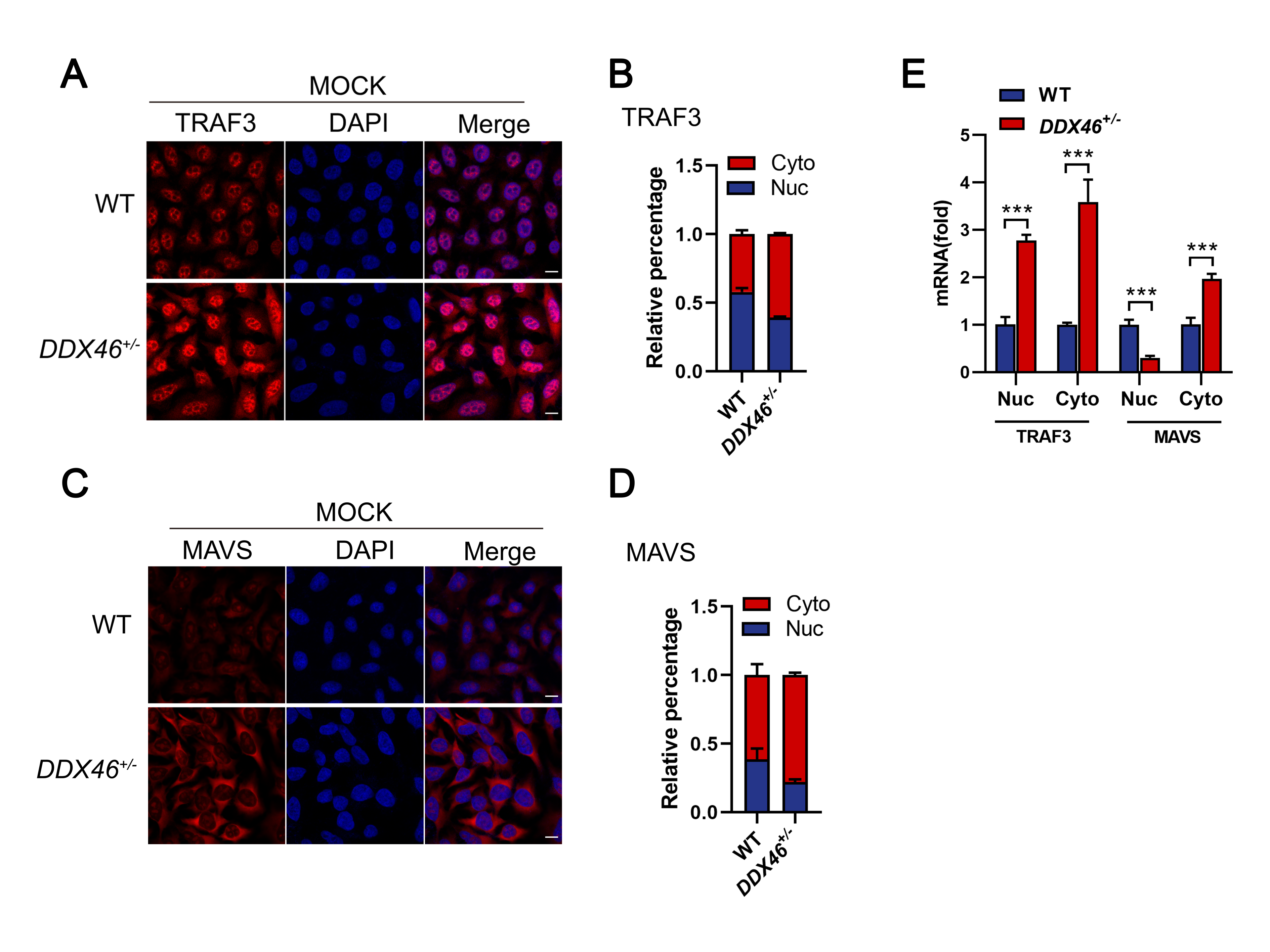

Supplement: Fig. S4 — DDX46 regulates the nuclear export of TRAF3 and MAVS transcripts. [file mbio.03519-25-s0004.tif]
