# Supplementary material for: Caspase-mediated DDX46 cleavage unchains antiviral immunity
Source: mBio. 2026 Mar 19;17(4):e03519-25. doi: 10.1128/mbio.03519-25 (PMC13059769; doi:10.1128/mbio.03519-25)
Supplement: Supplemental legends — Legends for all supplemental figures and tables. [file mbio.03519-25-s0005.docx]

# Supporting Information Legends

**Fig. S1 DDX46 negatively regulates the IFN-β signaling pathway.**

(A-B) DDX46 knockout cells were generated using a single sgRNA delivered via lentiviral CRISPR-Cas9. Following antibiotic selection, single-cell cloning yielded two subclones (DDX46#1 and DDX46#2). Knockout was validated by sequencing (A) and Western blot (B).

(C-F) WT and *DDX46*^+/-^ HeLa cells were mock-infected or infected with NDV(MOI=1) for 6, 12, 18, and 24 h. Protein levels of DDX46, p-TBK1, TBK1, and NDV-NP were analyzed by WB. β-actin served as the loading control (C). Extracellular NDV titers were determined by TCID50 (D). The intracellular mRNA levels of IFN-β (E) and IFIT-1 (F) were analyzed using qRT-PCR.

(G-J) WT and *DDX46*^+/-^ #2 HeLa cells were mock-infected or infected with VSV (MOI=1) for 6, 12, 18, and 24 h. Protein levels of DDX46, p-TBK1, TBK1, and VSV-G were analyzed by WB. β-actin served as the loading control (G). Extracellular VSV titers were determined by TCID_50_ (H). The intracellular mRNA levels of IFN-β (I) and IFIT-1 (J) were analyzed using qRT-PCR.

(K-M) Mouse macrophage RAW264.7 cells were transfected with siNC or siRNA targeting mouse DDX46 (siDdx46) for 48 h, then mock-infected or infected with VSV (MOI=1) for 12 and 24 h, followed by Western blot (WB) analysis of VSV G. β‑actin served as the loading control (K). The interference efficiency of DDX46 in mock-infected RAW264.7 cells was detected by qRT-PCR (L). The intracellular mRNA levels of IFN-β were analyzed using qRT-PCR (M).

Data are presented as means from three independent experiments. *P < 0.05, **P < 0.01, ***P < 0.001.

**Fig. S2 The cleavage of DDX46 by virus infection in various cell types**

1. C) A549 cells were mock-infected or infected with VSV (A), NDV (B) or HSV-1 (C) at an MOI of 1 for 6, 12, 18, and 24 h. Protein levels of VSV-G, NDV-NP, or HSV-1-gD were analyzed by WB. β-actin served as the loading control.

(D-F) H1299 cells were mock-infected or infected with VSV (D), NDV (E) or HSV-1 (F) at an MOI of 1 for 6, 12, 18, and 24 h. Protein levels of VSV-G, NDV-NP, or HSV-1-gD were analyzed by WB. β-actin served as the loading control.

**Fig. S3 DDX46 cleavage promotes IFN-β pathway early in viral infection.**

(A-D) *DDX46*^+/-^ cells stably expressing HA-tagged WT-DDX46 (OE-DDX46) and D226A-DDX46 (OE-D226A) were established. OE-DDX46 and OE-D226A cells were infected with VSV (MOI=1) for 6, 12 and 18 h. Protein levels of HA-DDX46 and VSV-G were analyzed by WB. β-actin served as the loading control (A). Extracellular VSV titers were determined by TCID_50_ (B). The intracellular mRNA levels of IFN-β (C) and IFIT-1 (D) were analyzed using qRT-PCR.

(E-G) Virus infection experiments were performed as in (A), except the cell harvest times were changed to 0, 3, 6, and 9 hpi. The intracellular mRNA levels of IFN-β (E), IFIT-1 (F) and VSV-G (G) were analyzed using qRT-PCR.

(H and I) OE-DDX46 and OE-D226A cells were transfected with poly(I:C). The cells were lysed at 0, 6 and12 hpt. The intracellular mRNA levels of IFN-β (H), IFIT-1 (I) were analyzed using qRT-PCR.

Data are presented as means from three independent experiments. *P < 0.05, **P < 0.01, ***P < 0.001.

**Fig. S4 DDX46 regulates the nuclear export of TRAF3 and MAVS transcripts.**

(A-D) WT and *DDX46*^+/-^ cells were fixed and subjected to FISH analysis using probes for TRAF3 (A and B) and MAVS (C and D). Nuclei were counterstained with DAPI. Quantification of the relative percentages of cells with nuclear or cytoplasmic TRAF3 (B) or MAVS (D) mRNA signal. Six randomly selected fields were analyzed in ImageJ

(E) WT and *DDX46*^+/-^ cells were harvested, and nuclear and cytoplasmic fractions were prepared using a nucleocytoplasmic isolation kit. The nuclear and cytoplasmic mRNA levels of TRAF3 and MAVS were analyzed using qRT-PCR.

Data are presented as means from three independent experiments. ***P < 0.001.

**Table S1 Primers used in this study**

**Table S2 Complete list of RBP hits and metrics from CRISPR library screening**
