# Supplementary material for: Caspase-mediated DDX46 cleavage unchains antiviral immunity
Source: mBio. 2026 Mar 19;17(4):e03519-25. doi: 10.1128/mbio.03519-25 (PMC13059769; doi:10.1128/mbio.03519-25)
Supplement: Table S1 — Primers used in this study. [file mbio.03519-25-s0006.docx]

Table 2 Primers used in this study

| Primers | Application | Sequence (5’-3’) |
| --- | --- | --- |
| FLAG-DDX46 F | Clone | taagcttgcggccgcgaattcATGGGTCGGGAGTCACGC |
| FLAG-DDX46 R |  | cctctagagtcgactggtaccgcTAAGACTTTGTATCTTCCTTTATTTGTTGG |
| FLAG-DDX46 △1-228 F |  | TAAGCTTGCGGCCGCGAATTCATGGATGCTTACATGGAAGAAGTGAAA |
| FLAG-DDX46 △1-228 R |  | CCTCTAGAGTCGACTGGTACCGCTAAGACTTTGTATCTTCCTTTATTTGTTGG |
| FLAG-DDX46 △229-402 F1 |  | CGCTAGCCTAAGATAAAAGATCCTCTATCAAG |
| FLAG-DDX46 △229-402 R1 |  | CATTATAGCAGGAATTAATGGATCTAACTC |
| FLAG-DDX46 △229-402 F2 |  | GAGTTAGATCCATTAATTCCTGCTATAATG |
| FLAG-DDX46 △229-402 R2 |  | CCTCTAGAGTCGACTGGTACCGCTAAGACTTTGTATCTTCCTTTATTTGTTGG |
| FLAG-DDX46 △403-591 F1 |  | TAAGCTTGCGGCCGCGAATTCATGGGTCGGGAGTCACGC |
| FLAG-DDX46 △403-591 R1 |  | TTGTTGCTCCACATCAGCTTGGGTTTGGAT |
| FLAG-DDX46 △403-591 F2 |  | ATCCAAACCCAAGCTGATGTGGAGCAACAA |
| FLAG-DDX46 △403-591 R2 |  | CCTCTAGAGTCGACTGGTACCGCTAAGACTTTGTATCTTCCTTTATTTGTTGG |
| FLAG-DDX46 △592-753 F1 |  | TAAGCTTGCGGCCGCGAATTCATGGGTCGGGAGTCACGC |
| FLAG-DDX46 △592-753 R1 |  | ATCTTTGAAATCACTTGAGCAAACCACACT |
| FLAG-DDX46 △592-753 F2 |  | AGTGTGGTTTGCTCAAGTGATTTCAAAGAT |
| FLAG-DDX46 △592-753 R2 |  | CCTCTAGAGTCGACTGGTACCGCTAAGACTTTGTATCTTCCTTTATTTGTTGG |
| FLAG-DDX46△754-1032 F1 |  | TAAGCTTGCGGCCGCGAATTCATGGGTCGGGAGTCACGC |
| FLAG-DDX46△754-1032 R1 |  | CCTCTAGAGTCGACTGGTACCGCCCACAGTTTCTCTAAATCAGGAGGTACTGC |
| FLAG-DDX46 Mid F |  | CGCGGAGTTCTACCGGCAGTGCAAATCCGTC |
| FLAG-DDX46 Mid R |  | CTGCCGGTAGAACTCCGCGAGGTCGTCCAGC |
| FLAG-DDX46 D147A F |  | AGAAAAAGCTGCTGGCAACTTTGACCAGAATAAGC |
| FLAG-DDX46 D147A R |  | TTGCCAGCAGCTTTTTCTTTTTCATCCTCCTTGTC |
| FLAG-DDX46 D152A F |  | TTTGCCCAGAATAAGCTGGAAGAAGAAATGAGAAAGC |
| FLAG-DDX46 D152A R |  | AGCTTATTCTGGGCAAAGTTGCCAGCATCTTT |
| FLAG-DDX46 D202-206A F |  | AGGCCGCTGCTGCTGCCGAAGATGATCCTG |
| FLAG-DDX46 D202-206A R |  | CGGCAGCAGCAGCGGCCTCTAAACTCCACTT |
| FLAG-DDX46 D208.209A F |  | GACGAAGCTGCTCCTGCAGAAGCTGAAAAG |
| FLAG-DDX46 D208.209A R |  | AGGAGCAGCTTCGTCATCATCATCGTCCTC |
| FLAG-DDX46 D202-206.208.209A F |  | GCCGAAGCTGCTCCTGCAGAAGCTGAAAAGGAGG |
| FLAG-DDX46 D202-206.208.209A R |  | TGCAGGAGCAGCTTCGGCAGCAGCAGCG |
| OE-DDX46-F |  | ttcaggtgtcgtgaagcggccgcATGTACCCATACGATGTTCCAGATTACGCTGGTCGGGAGTCACGCCAC |
| OE-DDX46-R |  | cgttaggggggggggtctagaTTAACCGTCATGGTCTTTGTAGTCTAAGACTTTGTATCTTCCTTTATTTGTTGGTTGGTATGAATT |
| si-DDX46 (Mouse) F |  | GCAGAGAAAUAGUUUGUGATT |
| si-DDX46 (Mouse) R |  | UCACAAACUAUUUCUCUGCTT |
| si-DDX46 (Human) F | Knockdown | GGUCUUCUUAAGGAUUUAATT |
| si-DDX46 (Human) R |  | UUAAAUCCUUAAGAAGACCTT |
| si-DHX58 (Human) F |  | GGGCUCUUGACCAAUGAAATT |
| si-DHX58 (Human) R |  | UUUCAUUGGUCAAGAGCCCTT |
| si-DDX41 (Human) F |  | CACCCAUCAAGAGCUUCAATT |
| si-DDX41 (Human) R |  | UUGAAGCUCUUGAUGGGUGTT |
| si-DDX60L (Human) F |  | GCGCUAACGACAGAUAUUATT |
| si-DDX60L (Human) R |  | UAAUAUCUGUCGUUAGCGCTT |
| si-DDX60 (Human) F |  | GCCUCCCAAAGCUGAUAAATT |
| si-DDX60 (Human) R |  | UUUAUCAGCUUUGGGAGGCTT |
| si-DDX25 (Human) F |  | GCAGCUAAUUCACUCUUAATT |
| si-DDX25 (Human) R |  | UUAAGAGUGAAUUAGCUGCTT |
| si-DDX51 (Human) F |  | CAUCUACACAGAUGCCACATT |
| si-DDX51 (Human) R |  | UGUGGCAUCUGUGUAGAUGTT |
| si-DDX5 (Human) F |  | GGUGGAUUUAAUACCUUUATT |
| si-DDX5 (Human) R |  | UAAAGGUAUUAAAUCCACCTT |
| si-DDX24 (Human) F |  | GAACCGUCGUCCUGAGAUUTT |
| si-DDX24 (Human) R |  | AAUCUCAGGACGACGGUUCTT |
| Human GAPDH F |  | GGAGCGAGATCCCTCCAAAAT |
| Human GAPDH R |  | GGCTGTTGTCATACTTCTCATGG |
| Human IFN-β F | qRT-PCR | ATGACCAACAAGTGTCTCCTCC |
| Human IFN-β R |  | GGAATCCAAGCAAGTTGTAGCTC |
| Human IFIT1 F |  | TTGATGACGATGAAATGCCTGA |
| Human IFIT1 R |  | CAGGTCACCAGACTCCTCAC |
| Human U2 sn-F |  | CATCGCTTCTCGGCCTTTTG |
| Human U2 sn-R |  | TGGAGGTACTGCAATACCAGG |
| Human MAVS-F |  | GCATCAGGAGCAGGACACAGAAC |
| Human MAVS-R |  | TGGAAGGAGACAGATGGAGACACAG |
| Human TRAF3-F |  | ACATCCGCCTAGCCGACATGG |
| Human TRAF3-R |  | CTGCTTCCGCCGCTTGTAGTC |
| VSV-G-F |  | AGGCACAGCCATACAAGTCAAA |
| VSV-G-R |  | TTTGGAAGCATGACACATCCA |

|  |
| --- |
